# Deletion of *RFX6* impairs iPSC-derived islet organoid development and survival, with no impact on PDX1^+^/NKX6.1^+^ progenitors

**DOI:** 10.1007/s00125-024-06232-2

**Published:** 2024-07-30

**Authors:** Noura Aldous, Ahmed K. Elsayed, Bushra Memon, Sadaf Ijaz, Sikander Hayat, Essam M. Abdelalim

**Affiliations:** 1 College of Health and Life Sciences, Hamad Bin Khalifa University (HBKU), Qatar Foundation (QF), Education City, Doha, Qatar; 2 Laboratory of Pluripotent Stem Cell Disease Modeling, Translational Medicine Department, Research Branch, Sidra Medicine, Doha, Qatar; 3 Diabetes Research Center, Qatar Biomedical Research Institute (QBRI), Hamad Bin Khalifa University (HBKU), Qatar Foundation (QF), Doha, Qatar; 4 Stem Cell Core, Qatar Biomedical Research Institute (QBRI), Hamad Bin Khalifa University (HBKU), Qatar Foundation (QF), Doha, Qatar; 5 Department of Medicine 2 (Nephrology, Rheumatology, Clinical Immunology and Hypertension), RWTH Aachen University, Medical Faculty, Aachen, Germany

**Keywords:** Diabetes, Endocrine specification, Islet organoids, Pancreatic hypoplasia, Pancreatic progenitors, Transcription factors

## Abstract

**Aims/hypothesis:**

Homozygous mutations in *RFX6* lead to neonatal diabetes accompanied by a hypoplastic pancreas, whereas heterozygous mutations cause MODY. Recent studies have also shown *RFX6* variants to be linked with type 2 diabetes. Despite *RFX6*’s known function in islet development, its specific role in diabetes pathogenesis remains unclear. Here, we aimed to understand the mechanisms underlying the impairment of pancreatic islet development and subsequent hypoplasia due to loss-of-function mutations in *RFX6*.

**Methods:**

We examined regulatory factor X6 (RFX6) expression during human embryonic stem cell (hESC) differentiation into pancreatic islets and re-analysed a single-cell RNA-seq dataset to identify RFX6-specific cell populations during islet development. Furthermore, induced pluripotent stem cell (iPSC) lines lacking RFX6 were generated using CRISPR/Cas9. Various approaches were then employed to explore the consequences of RFX6 loss across different developmental stages. Subsequently, we evaluated transcriptional changes resulting from RFX6 loss through RNA-seq of pancreatic progenitors (PPs) and endocrine progenitors (EPs).

**Results:**

RFX6 expression was detected in PDX1^+^ cells in the hESC-derived posterior foregut (PF). However, in the PPs, RFX6 did not co-localise with pancreatic and duodenal homeobox 1 (PDX1) or NK homeobox 1 (NKX6.1) but instead co-localised with neurogenin 3, NK2 homeobox 2 and islet hormones in the EPs and islets. Single-cell analysis revealed high *RFX6* expression levels in endocrine clusters across various hESC-derived pancreatic differentiation stages. Upon differentiating iPSCs lacking RFX6 into pancreatic islets, a significant decrease in PDX1 expression at the PF stage was observed, although this did not affect PPs co-expressing PDX1 and NKX6.1. RNA-seq analysis showed the downregulation of essential genes involved in pancreatic endocrine differentiation, insulin secretion and ion transport due to RFX6 deficiency. Furthermore, RFX6 deficiency resulted in the formation of smaller islet organoids due to increased cellular apoptosis, linked to reduced catalase expression, implying a protective role for RFX6. Overexpression of RFX6 reversed defective phenotypes in *RFX6*-knockout PPs, EPs and islets.

**Conclusions/interpretation:**

These findings suggest that pancreatic hypoplasia and reduced islet cell formation associated with RFX6 mutations are not due to alterations in PDX1^+^/NKX6.1^+^ PPs but instead result from cellular apoptosis and downregulation of pancreatic endocrine genes.

**Data availability:**

RNA-seq datasets have been deposited in the Zenodo repository with accession link (DOI: https://doi.org/10.5281/zenodo.10656891).

**Graphical Abstract:**

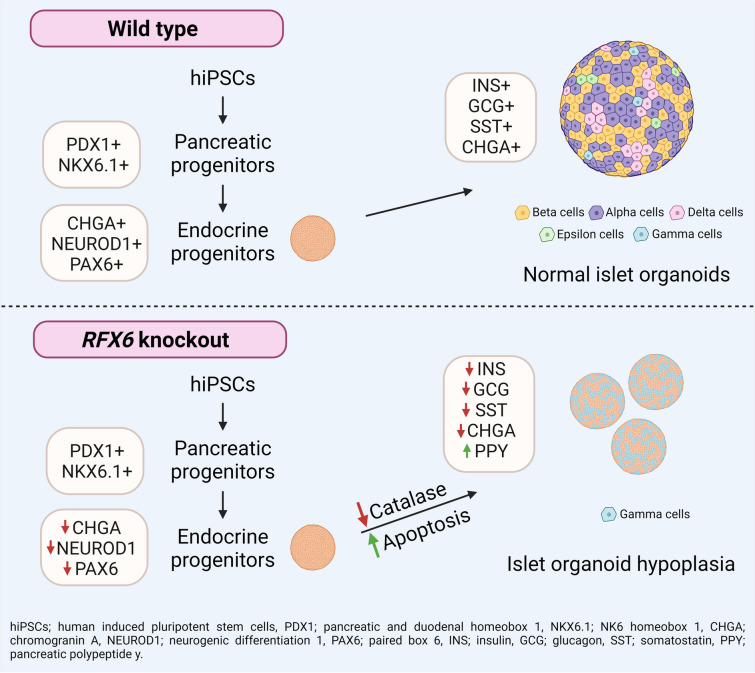

**Supplementary Information:**

The online version of this article (10.1007/s00125-024-06232-2) contains peer-reviewed but unedited supplementary material.


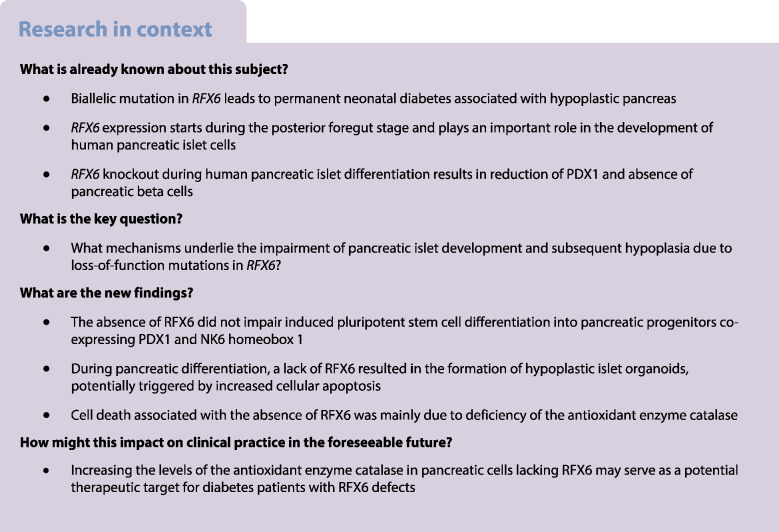


## Introduction

Regulatory factor X6 (RFX6), a critical transcription factor, is crucial for islet cell development and function. Homozygous mutations in *RFX6* cause Mitchell–Riley syndrome (MRS), which is characterised by severe neonatal diabetes associated with hypoplastic pancreas, and intestinal atresia [1–5]. It has been suggested that this form of diabetes is attributed to the overall impairment of pancreatic islet development and function, including a reduction in insulin (INS) production by beta cells [1, 6]. In contrast, heterozygous mutations result in mild MODY [7–9], where defective INS secretion occurs despite normal islet development [4, 10]. Thus, it appears that RFX6 governs both islet development and INS production, albeit through different mechanisms. Knocking down RFX6 in human EndoC-βH1 cells alters *INS* mRNA levels [4]. Furthermore, genome-wide association studies link *RFX6* variants to type 2 diabetes [11] and analysis of multiomics data from individuals with type 2 diabetes reveals genetic regions enriched with RFX-binding motifs [12].

Mouse studies reveal that *Rfx6* expression initiates in gut endoderm, undergoes a developmental transition and becomes restricted to the endocrine lineage within the pancreas, persisting in adult islet cells. The significance of *Rfx6* in islet cell development is evident across various species [1, 2, 6, 13, 14]. In mice with *Rfx6* gene deficiency, all endocrine cells are absent, except for polypeptide-secreting cells, resulting in diabetes and early postnatal death, limiting the exploration of its role in beta cell function and INS production [1, 6]. In adult beta cells, the loss of *Rfx6* results in glucose intolerance, impaired glucose sensing and defective INS secretion [10]. Moreover, RFX6 is expressed in gastric inhibitory polypeptide (GIP)-positive enteroendocrine K cells, regulating GIP promoter activity [15]. Furthermore, a recent study demonstrated that the loss of *Rfx6* function in ex vivo mouse intestinal organoids reduces enteroendocrine cells (EECs) [16].

Mutations in *RFX6* have led to different types of diabetes [1], raising questions about its importance in pancreatic islet development and function. Understanding its role could lead to new diabetes treatments. While previous research shows RFX6 is crucial for islet development and glucose regulation, its exact involvement in diabetes is unclear. This study used CRISPR/Cas9 to generate induced pluripotent stem cells (iPSCs) lacking RFX6, then differentiating them into islet cells. We aimed to investigate the impact of RFX6 loss at different developmental stages to determine the role of RFX6 in endocrine pancreatic development and islet survival.

## Methods

### Culture of human embryonic stem cells and induced pluripotent stem cells

HA-RFX6 tagged H9 human embryonic stem cell (hESC) line (*RFX6*^HA/HA^ H9-hESCs) and its control, H9-hESCs, were obtained from N. R. Dunn (A*STAR, Singapore). Wild-type (WT) induced pluripotent stem cells (iPSCs) generated in our laboratory were used [17, 18]. All cells were cultured in mTeSR Plus medium (Stem Cell Technologies, Canada) on Matrigel-coated dishes (Corning, USA) [19, 20].

### Differentiation of hESCs and iPSCs into pancreatic islets

hESC/iPSC lines were differentiated in vitro into pancreatic progenitors (PPs) using our protocol [21]. The protocol of Veres et al was adapted for further differentiation into pancreatic islet cells [22]. See electronic supplementary material (ESM) Methods and ESM Table 1 for details.

### Generation of *RFX6*-knockout iPSCs

We generated two *RFX6*-knockout (KO) human iPSC lines (RFX6 KO1 and RFX6 KO2) from WT iPSCs using Lipofectamine 3000 Transfection Reagent (ThermoFisher Scientific, Waltham, MA, USA) for the GFP-tagged plasmid vector expressing spCas9 and gRNA. Cells were sorted based on GFP expression at 48 h post-transfection.

### Paraffin embedding and immunofluorescence

We performed immunostaining as reported previously [19] and used the paraffin embedding technique for 3D pancreatic islet organoids following established protocols [23, 24]. See ESM Methods and ESM Table 2 for details.

### Flow cytometry, western blotting, PCR and RT-PCR

Flow cytometry, western blotting, PCR and RT-PCR were conducted as reported previously. See ESM Methods and ESM Tables 2, 3 for details.

### Single-cell and RNA-seq analyses

We used the GSE202497 dataset for single-cell analysis (https://www.ncbi.nlm.nih.gov/geo/query/acc.cgi?acc=GSE202497) [25]. Further details on single-cell and RNA-seq analyses are available in ESM Methods.

### Apoptosis and proliferation assays

The apoptosis and proliferation assays were performed, following previously established protocols [26] (see ESM Methods).

### *RFX6* overexpression

RFX6 overexpression was induced as previously reported [26] (see ESM Methods).

### Analysis of protein–protein interaction networks associated with *CAT*

To explore *CAT* interactions and predict functional associations, we employed the STRING database (https://string-db.org) [27].

### Statistical analysis

At least three biological replicates were used in most experiments while statistical analysis was done using unpaired two-tailed Student’s *t* test on Prism version 8 (GraphPad Software, Boston, MA, USA, www.graphpad.com), with data represented as mean ± SD.

## Results

### Temporal and cell-specific expression of RFX6 during pancreatic islet differentiation

**Fig. 1:**
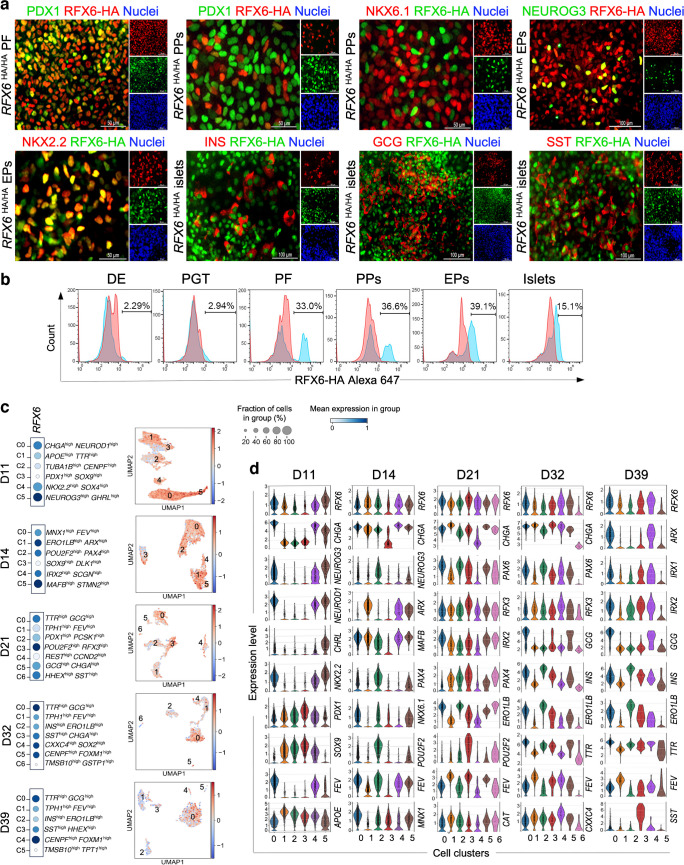
Timeline expression and single-cell analysis of RFX6 throughout the differentiation of hESCs into various stages of pancreatic development. (**a**) Immunostaining showing the expression of RFX6 during differentiation of hESC-H9 into pancreatic islets. (**b**) Flow cytometric quantification of RFX6 expression during different stages of differentiation. (**c**) Dot plots and feature plots illustrating *RFX6* expression across distinct cell clusters. Each dot’s colour and size correspond to the expression level and the percentage of cells expressing the *RFX6* gene. (**d**) The violin plots illustrate the expression distributions of key genes across various clusters at distinct stages of hESC differentiation into pancreatic islets: day 11 (D11); day 14 (D14); day 21 (D21); day 32 (D32); and day 39 (D39). DE, definitive endoderm; PGT, primitive gut tube. Scale bar, 100 µm

To examine RFX6 expression during pancreatic islet differentiation, we tracked its levels in hESC-H9 cells at various differentiation stages (ESM Fig. 1a). Due to the lack of a specific antibody for RFX6 immunostaining, we used a modified *RFX6*^HA/HA^ hESC-H9 line, incorporating a triplicated haemagglutinin (HA) epitope to enable RFX6 protein detection [28]. Initially, RFX6 immunoreactivity was absent during the definitive endoderm and primitive gut tube stages (ESM Fig. 1b). However, robust expression emerged in the posterior foregut (PF), largely co-localised with pancreatic and duodenal homeobox 1 (PDX1) (Fig. 1a). Interestingly, while RFX6 persisted in the PP stage, it did not co-localise with PDX1 or NK6 homeobox 1 (NKX6.1) (Fig. 1a). During the endocrine progenitor (EP) stage, RFX6 co-localised with neurogenin 3 (NEUROG3) and NK2 homeobox 2 (NKX2.2), the pancreatic endocrine markers (Fig. 1a). In the islet cell stage, RFX6 co-expressed with INS, glucagon (GCG) and somatostatin (SST) (Fig. 1a). Flow cytometry analysis confirmed high RFX6 levels from the PF stage onwards, peaking at the EP stage (Fig. 1b). These findings suggest that RFX6 may not be indispensable for PDX1^+^/NKX6.1^+^ PPs.

To delve deeper into identifying specific cell populations expressing *RFX6*, we re-analysed the recently published single-cell (scRNA-seq) datasets of PPs at day 11 (D11), EPs at day 14 (D14), immature islets at day 21 (D21) and maturing islets at days 32 and 39 (D32 and D39), derived from hESCs [25]. We used unsupervised clustering to create 2D visualisations using uniform manifold approximation and projection (UMAP) plots and identified multiple cell populations at each differentiation stage (Fig. 1c, d and ESM Fig. 2). At D11, six cell clusters were identified, with three showing high expression levels of pancreatic endocrine markers. RFX6 was mainly expressed in these clusters, with the highest level in C5, distinguished by *NEUROG3*^high^/GHRL^high^, which also expressed high levels of other endocrine markers such as *PAX4*, *INSM1*, *KCNK17*, *NKX2.2* and *SOX4*. Moderate RFX6 expression was observed in two additional endocrine clusters (C0 and C4), characterised by *CHGA*^high^/*NEUROD1*^high^ and *NKX2.2*^high^/*SOX4*^high^, respectively. Low *RFX6* expression was seen in C1 (*APOE*^high^/*TTR*^high^) and C2 (proliferation cluster; *TUBA1B*^high^/*CENPF*^high^). Almost no expression was detected in C3, identified by *PDX1*^high^/*SOX9*^high^ (Fig. 1c, d and ESM Fig. 2). These findings strongly indicate that RFX6 expression in PPs is confined to endocrine cell populations.

At D14, we identified six clusters, with the highest expression of RFX6 observed in the endocrine cluster C5, marked by *MAFB*^high^/*STMN*2^high^; this cluster also showed elevated levels of essential endocrine markers such as *INS*,* GCG* and *SLC30A8* (Fig. 1c, d and ESM Fig. 2). Another endocrine cluster, C1 (*ERO1LB*^high^/*ARX*^high^), displayed high RFX6 expression levels. In addition, a moderate *RFX6* level was detected in C2 (*POU2F2*^high^/*PAX4*^high^), C0 (*MNX1*^high^/*FEV*^high^) and C4 (*IRX2*^high^/*SCGN*^high^). The lowest RFX6 expression was seen in C3 (PP cluster; *SOX9*^high^/*DLK1*^high^), which also expressed high levels of *PDX1*,* HNF1B*,* GATA4*, *TCF7L2* and *CCND2*. At D21, the highest expression of RFX6 was seen in C3 (*POU2F2*^high^/*RFX3*^high^), while a moderate expression was seen in C0 (*TTR*^high^/*GCG*^high^), C5 (*GCG*^high^/*CHGA*^high^) and C6 (delta cell cluster; *HHEX*^high^/*SST*^high^). Reduced expression was observed in C1 (*TPH1*^high^/*FEV*^high^), previously identified as a specific enterochromaffin progenitor population [29], and in C4 (proliferation cluster; *REST*^high^/*CCND2*^high^) (Fig. 1c, d and ESM Fig. 2).

At D32, the most significant RFX6 expression was seen in C0 (*TTR*^high^/*GCG*^high^) (Fig. 1c), which also expressed high levels of *CHGA*,* IRX2*, and *ARX*, suggesting an alpha cell fate. Moderate RFX6 expression was seen in C4 (*CXXC4*^high^/*SOX2*^high^) and C5 (proliferation cluster; *CENPF*^high^/*FOXM1*^high^), while lower levels were seen in C1 (*TPH1*^high^/*FEV*^high^) and C2 (*INS*^high^/*ERO1LB*^high^). *ERO1LB* (also known as *ERO1B*) is known as a gene specifically associated with pancreatic beta cells [30]. No expression was observed in C6 (*TMSB10*^high^/*GSTP1*^high^). At D39, the highest RFX6 expression was seen in C4 (proliferation cluster; *CENPF*^high^/*FOXM1*^high^), with moderate expression in C0 (*TTR*^high^/*GCG*^high^). Lower expression was seen in C3 (*SST*^high^/*HHEX*^high^), C1 (*TPH1*^high^/*FEV*^high^) and C2 (*INS*^high^/*ERO1LB*^high^), while no expression was seen in C5 (*TMSB10*^high^/*TPT1*^high^) (Fig. 1c, d and ESM Fig. 2). Taken together, these findings indicate that RFX6 is mainly expressed in pancreatic endocrine cell populations across various stages and is not expressed in *PDX1*^+^ cell populations within PPs.

### Depletion of RFX6 diminishes PDX1 expression in the PF and does not affect PDX1^+^/NKX6.1^+^ PPs

**Fig. 2:**
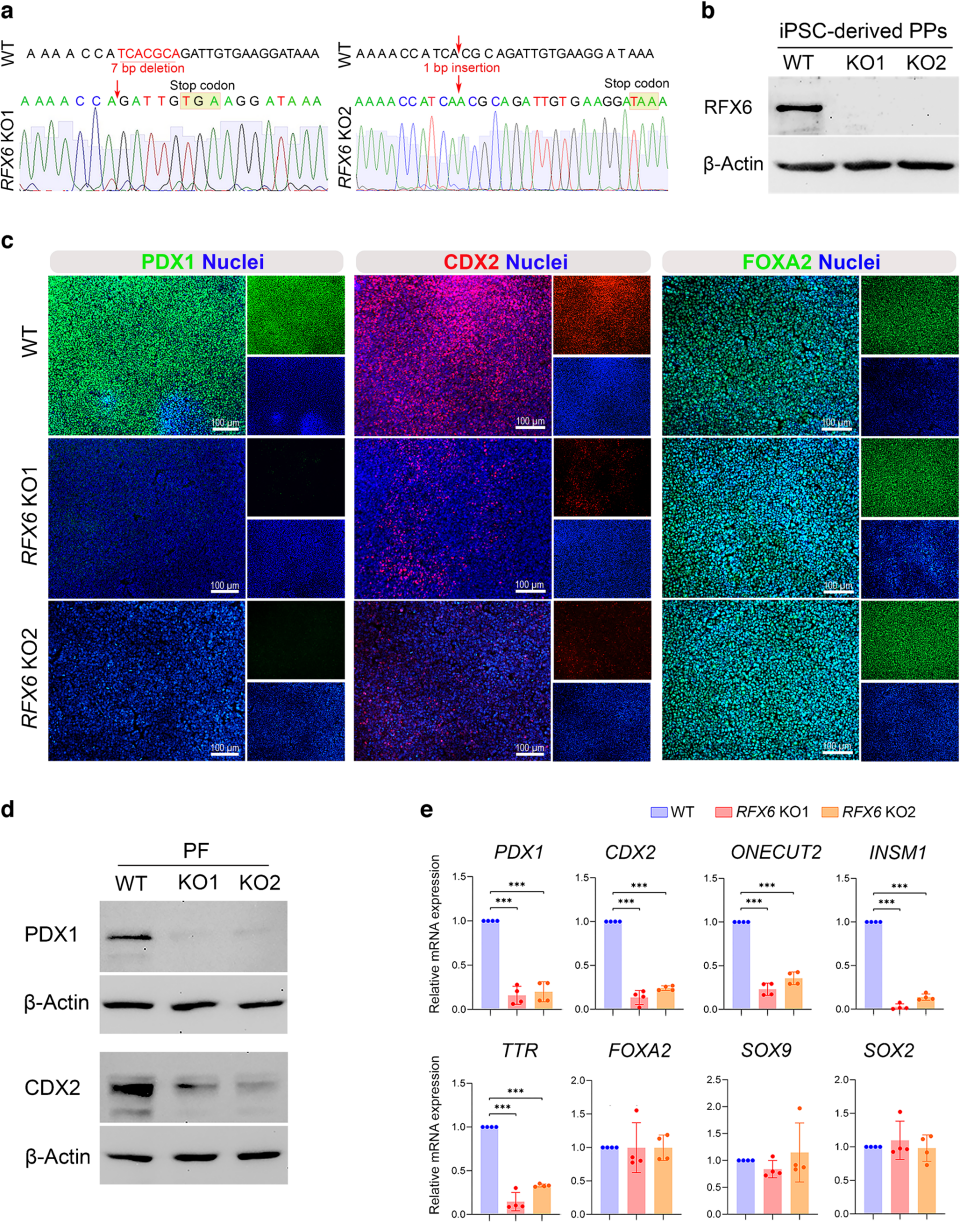
Loss of *RFX6* reduces PDX1 and CDX2 expression in iPSC-derived PF. (**a**) DNA sequence confirmation of frameshift mutations in isogenic KO iPSC clones compared with WT iPSCs. (**b**) Western blot analysis confirming the absence of RFX6 protein in PPs derived from *RFX6* KO iPSC lines. (**c**) Immunofluorescence images showing the expression of PDX1, CDX2 and FOXA2 in PPs derived from WT iPSCs and *RFX6* KO iPSCs. (**d**) Western blot analysis showing the expression of PDX1 and CDX2 in *RFX6* KO PF compared with WT PF. (**e**) RT-qPCR analysis showing the mRNA expression of PF markers *PDX1*, *CDX2*, *ONECUT2*, *INSM1*, *TTR*, *FOXA2*, *SOX9* and *SOX2* in *RFX6* KO PF relative to WT control PF (*n*=4). The data are presented as mean ± SD. ***p<0.001. Scale bar, 100 µm

To explore RFX6’s contribution to human pancreatic development and the generation of pancreatic islet cells, biallelic *RFX6* mutant human iPSC lines (referred to as *RFX6* KO iPSCs) were established using the CRISPR/Cas9 system. Mutations were introduced into the WT iPSC line generated in our laboratory [18] using a gRNA that targeted exon 2 of *RFX6*. The mutations were validated through Sanger sequencing and were anticipated to induce a frameshift resulting in the formation of premature stop codons preventing RFX6 protein translation (Fig. 2a). The absence of RFX6 protein expression was confirmed in PPs derived from *RFX6* KO cell lines compared with WT controls using western blot analysis (Fig. 2b). All iPSC lines expressed pluripotency markers *OCT4* (also known as *POU5F1*), *NANOG*, *SOX2*, *SSEA4*, *TRA-1-60*, *TRA-81*, *C-MYC* (also known as *MYC*), *KLF4*, *REX1* (also known as *ZFP42*), *DPPA4* and *TERT* (ESM Fig. 3a, b). Moreover, they have been verified to maintain normal karyotypes consistent with the parental line and are free from mycoplasma (ESM Fig. 3c, d).

**Fig. 3:**
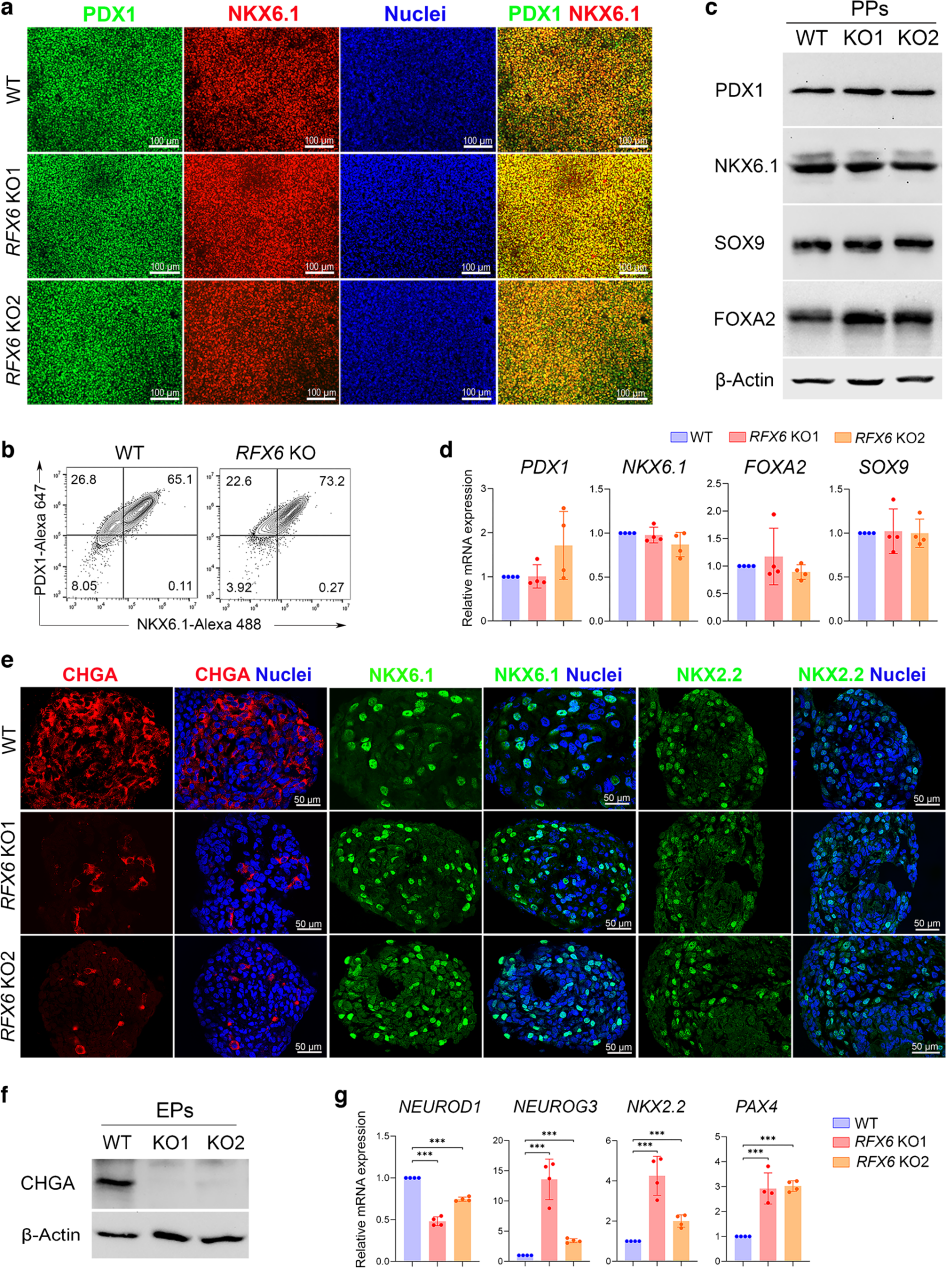
Impact of *RFX6* depletion on the expression of crucial pancreatic progenitor and endocrine progenitor markers. (**a**, **b**) Immunofluorescence staining (**a**) and flow cytometry analysis (**b**) showing the co-expression of PDX1 and NKX6.1 in PPs derived from WT iPSCs and *RFX6* KO iPSCs. PDX1^+^/NKX6.1^+^ cells are shown in the upper right quadrant in (**b**). (**c**) Western blot analysis showing the protein expression of PDX1, NKX6.1, SOX9 and FOXA2 in *RFX6* KO PPs compared with WT PPs. (**d**) RT-qPCR analysis showing the mRNA expression of PP markers *PDX1*, *NKX6.1*, *FOXA2* and *SOX9* in *RFX6* KO PPs relative to WT PPs (*n*=4). (**e**) Immunofluorescence staining showing the expression of CHGA, NKX6.1 and NKX2.2 in EPs derived from WT iPSCs and *RFX6* KO iPSCs. (**f**) Western blot analysis showing the expression of CHGA in *RFX6* KO EPs compared with WT EPs. (**g**) RT-qPCR analysis showing the mRNA expression of EP markers *NEUROD1*, *NEUROG3*, *NKX2.2* and *PAX4* in *RFX6* KO EPs relative to WT EPs (*n*=4). The data are presented as mean ± SD. ***p<0.001. Scale bar, 50 μm (**e**) or 100 µm (**a**)

Next, we examined the effect of *RFX6* loss on pancreatic differentiation. Immunostaining and western blotting showed reduced PDX1 and caudal type homeobox 2 (CDX2) protein levels in *RFX6*-deficient PF cells, while forkhead box A2 (FOXA2) remained unchanged (Fig. 2c, d). qPCR analysis revealed significant downregulation in the mRNA expression of *PDX1*, *CDX2*, *ONECUT2*, *INSM1* and *TTR* in *RFX6* KO PF compared with WT PF (Fig. 2e). In contrast, *FOXA2*, *SOX9* and *SOX2* expression remained unaffected (Fig. 2e). Despite the dramatic reduction in PDX1 expression during the PF stage, *RFX6*-deficient iPSCs were able to produce PPs and co-expressed PDX1 and NKX6.1 like controls (Fig. 3a–d and ESM Fig. 4). Other PP markers, including SRY-box transcription factor (SOX)9 and FOXA2, remained unchanged, as evidenced by western blotting and RT-qPCR (Fig. 3c, d). These results suggest that RFX6 does not play a significant role in the formation of PDX1^+^/NKX6.1^+^ cells during the PP stage.

At the EP stage, we found that there was a dramatic reduction of the pan endocrine marker, CHGA (Fig. 3e, f), with no significant change in NKX6.1 and NKX2.2 expression (Fig. 3e). RT-qPCR analysis showed significant reduction in the expression of *NEUROD1*, while the expression of *NEUROG3*,* NKX2.2*, and *PAX4* were significantly increased in EPs lacking *RFX6* compared with WT controls (Fig. 3g).

### Deletion of *RFX6* leads to large-scale transcriptomic alterations associated with pancreatic endocrine specification in PPs and EPs

**Fig. 4:**
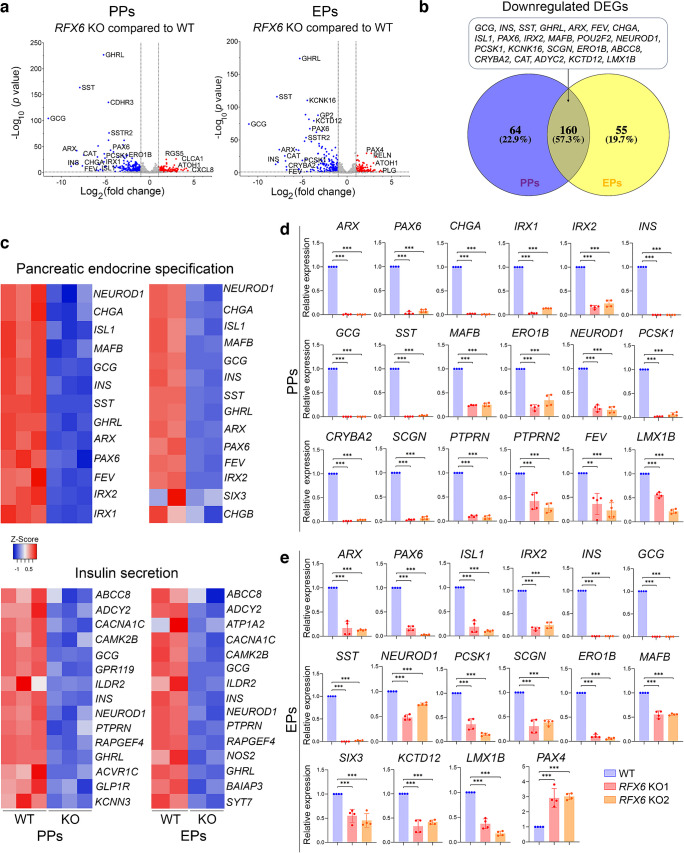
Impact of *RFX6* loss on transcriptomic profiles of iPSC-derived PPs and EPs. Bulk RNA-seq analysis was performed on PPs (*n*=3) and EPs (*n*=2) derived from *RFX6* KO iPSCs and WT iPSCs. (**a**) Volcano plots display the DEGs in *RFX6* KO PPs and *RFX6* KO EPs compared with their WT controls. Downregulated genes are represented by blue dots, while upregulated genes are depicted by red dots. (**b**) Venn diagram illustrating the intersection of downregulated DEGs in *RFX6* KO PPs and *RFX6* KO EPs. Note that most of those DEGs are endocrine pancreatic genes. (**c**) Heatmap of *z* score value of pancreatic endocrine and INS secretion genes downregulated in *RFX6* KO PPs and *RFX6* KO EPs compared with WT PPs and WT EPs, respectively. (**d**, **e**) RT-qPCR validation of the DEGs in PPs (**d**) and EPs (**e**) derived from two different KO iPSC lines (*n*=4). The data are presented as mean ± SD. **p<0.01, ***p<0.001

For comprehensive understanding of the transcriptomic changes between *RFX6* KO and WT cells, RNA-seq was performed on PPs and EPs. Our transcriptome analysis on iPSC-derived PPs detected 392 differentially expressed genes (DEGs) significantly affected by *RFX6* deletion. Among these DEGs, 223 genes were significantly downregulated (log_2_ fold change < −1.0, *p*<0.05), while 169 genes were significantly upregulated (log_2_ fold change >1.0, *p*<0.05) in *RFX6* KO PPs compared with WT PPs (Fig. 4a and ESM Fig. 5a). At the EP stage, we identified 325 DEGs significantly impacted by the deletion of *RFX6*, with 215 of these genes being significantly downregulated (log_2_ fold change < −1.0, *p*<0.05) and 110 genes being significantly upregulated (log_2_ fold change >1.0, *p*<0.05) in *RFX6* KO EPs compared with WT EPs (Fig. 4a and ESM Fig. 5b). Interestingly, 160 of the downregulated DEGs, comprising 57.3%, were found in both PPs and EPs (Fig. 4b), with most of these genes known to be associated with pancreatic endocrine development. The Gene Ontology (GO) of the downregulated DEGs in PPs and EPs displayed enriched genes linked to pancreatic endocrine development, INS secretion regulation, regulation of ion transmembrane transport and negative regulation of cell apoptosis (Fig. 4c and ESM Fig. 5c, d), whereas the upregulated DEGs showed GO enrichment in genes linked to lipid metabolism and nervous system development (data not shown). At the PP stage, the RT-qPCR validation analysis confirmed a significant decrease in the expression of endocrine genes including *ARX*, *PAX6*, *CHGA*, *IRX1*, *IRX2*, *INS*, *GCG*, *SST*, *MAF1B*, *ERO1B (ERO1LB)*, *NEUROD1*, *PCSK1*, *CRYBA2*, *SCGN*, *PTPRN*, *PRPRN2*, *FEV* and *LMX1B* in *RFX6* KO PPs compared with WT PPs (Fig. 4d, Table 1). Furthermore, at the EP stage, the RT-qPCR revealed significant decrease in the expression of endocrine genes including *ARX*, *PAX6*, *ISL1*, *IRX2*, *INS*, *GCG*, *SST*, *NEUROD1*, *PCSK1*, *SCGN*, *ERO1B*, *MAFB*, *SIX3*, *KCTD12* and *LMX1B* in *RFX6* KO EPs compared with WT EPs (Fig. 4e; Table 1).

**Table 1:** Key downregulated DEGs associated with pancreatic endocrine development and function in PPs and EPs lacking *RFX6* (log_2_ fold change < –1, *p*<0.05)

PPs			EPs		
Gene	Log_2_ FC	*p* value	Gene ID	Log_2_ FC	*p* value
*GCG*	−11.470	3.56 × 10^−105^	*GCG*	−11.002	6.40 × 10^−75^
*INS*	−8.409	2.04 × 10^−18^	*INS*	−7.747	1.05 × 10^−16^
*SST*	−7.909	1.29 × 10^−164^	*SST*	−7.860	1.42 × 10^−116^
*GHRL*	−5.224	1.00 × 10^−227^	*GHRL*	−5.303	8.81 × 10^−175^
*ARX*	−8.255	4.47 × 10^−43^	*ARX*	−7.600	5.66 × 10^−36^
*CHGA*	−5.818	4.65 × 10^−17^	*CHGA*	−6.103	0.00
*ISL1*	−5.681	2.47 × 10^−16^	*ISL1*	−5.418	9.30 × 10^−7^
*PAX6*	−4.434	6.02 × 10^−44^	*PAX6*	−4.172	6.96 × 10^−68^
*MAFB*	−2.653	4.23 × 10^−33^	*MAFB*	−2.863	1.70 × 10^−38^
*NEUROD1*	−2.944	1.97 × 10^−11^	*NEUROD1*	−3.148	4.23 × 10^−45^
*FEV*	−7.615	1.13 × 10^−13^	*FEV*	−6.774	3.32 × 10^−5^
*IRX2*	−3.056	3.41 × 10^−33^	*IRX2*	−2.881	1.92 × 10^−20^
*IRX1*	−5.180	1.25 × 10^−21^	*SIX3*	−1.056	2.26 × 10^−2^
*ABCC8*	−1.908	1.26 × 10^−6^	*ABCC8*	−1.540	5.13 × 10^−4^
*ADCY2*	−2.396	1.37 × 10^−10^	*ADCY2*	−2.238	4.23 × 10^−9^
*CACNA1C*	−2.398	3.72 × 10^−18^	*CACNA1C*	−2.100	2.73 × 10^−12^
*CAMK2B*	−2.335	7.84 × 10^−11^	*CAMK2B*	−2.689	2.18 × 10^−15^
*ILDR2*	−2.268	2.90 × 10^−8^	*ILDR2*	−2.602	2.67 × 10^−14^
*PTPRN*	−2.724	3.36 × 10^−6^	*PTPRN*	−3.444	1.58 × 10^−62^
*RAPGEF4*	−1.470	5.63 × 10^−18^	*RAPGEF4*	−1.558	3.86 × 10^−13^
*ACVR1C*	−1.778	1.46 × 10^−6^	*NOS2*	−4.068	1.53 × 10^−05^
*GLP1R*	−1.185	6.04 × 10^−7^	*BAIAP3*	−1.497	1.76 × 10^−16^
*GPR119*	−5.318	1.72 × 10^−4^	*ATP1A2*	−2.165	2.18 × 10^−4^
*CACNA1C*	−2.398	3.72 × 10^−18^	*CACNA1C*	−2.100	2.73 × 10^−12^
*CACNA2D1*	−2.013	2.16 × 10^−12^	*CACNA2D1*	−2.294	9.37 × 10^−33^
*CACNG7*	−1.268	3.05 × 10^−2^	*CACNG7*	−1.889	2.50 × 10^−4^
*CACNA1A*	−1.864	1.70 × 10^−12^	*CACNA1A*	−2.001	2.16 × 10^−18^
*CACNA1B*	−1.351	6.87 × 10^−7^	*CACNA1B*	−1.460	8.02 × 10^−7^
*KCNJ6*	−2.440	1.96 × 10^−10^	*KCNJ6*	−2.626	6.00 × 10^−13^
*KCNK16*	−3.887	1.24 × 10^−10^	*KCNK16*	−4.462	5.35 × 10^−111^
*KCNK17*	−1.774	4.11 × 10^−8^	*KCNK17*	−1.698	2.93 × 10^−17^
*KCNC4*	−1.415	2.00 × 10^−6^	*KCNC4*	−1.528	5.24 × 10^−5^
*KCND3*	−1.548	1.05 × 10^−15^	*KCND3*	−1.654	2.84 × 10^−12^
*KCNH6*	−1.414	1.63 × 10^−2^	*KCNH6*	−2.025	3.05 × 10^−21^
*SCN3A*	−1.766	7.56 × 10^−7^	*SCN3A*	−2.292	6.99 × 10^−14^
*SCN7A*	−1.370	4.49 × 10^−6^	*KCNV1*	−1.018	3.60 × 10^−5^
*KCNN3*	−2.817	9.59 × 10^−9^	*SYT7*	−1.192	1.52 × 10^−14^
*KCNJ5*	−3.003	4.47 × 10^−12^	*CHGB*	−1.212	6.04 × 10^−5^
*KCNK10*	−2.476	8.32 × 10^−6^			

FC, fold change

### *RFX6* loss correlates with the generation of smaller pancreatic islet organoids

**Fig. 5:**
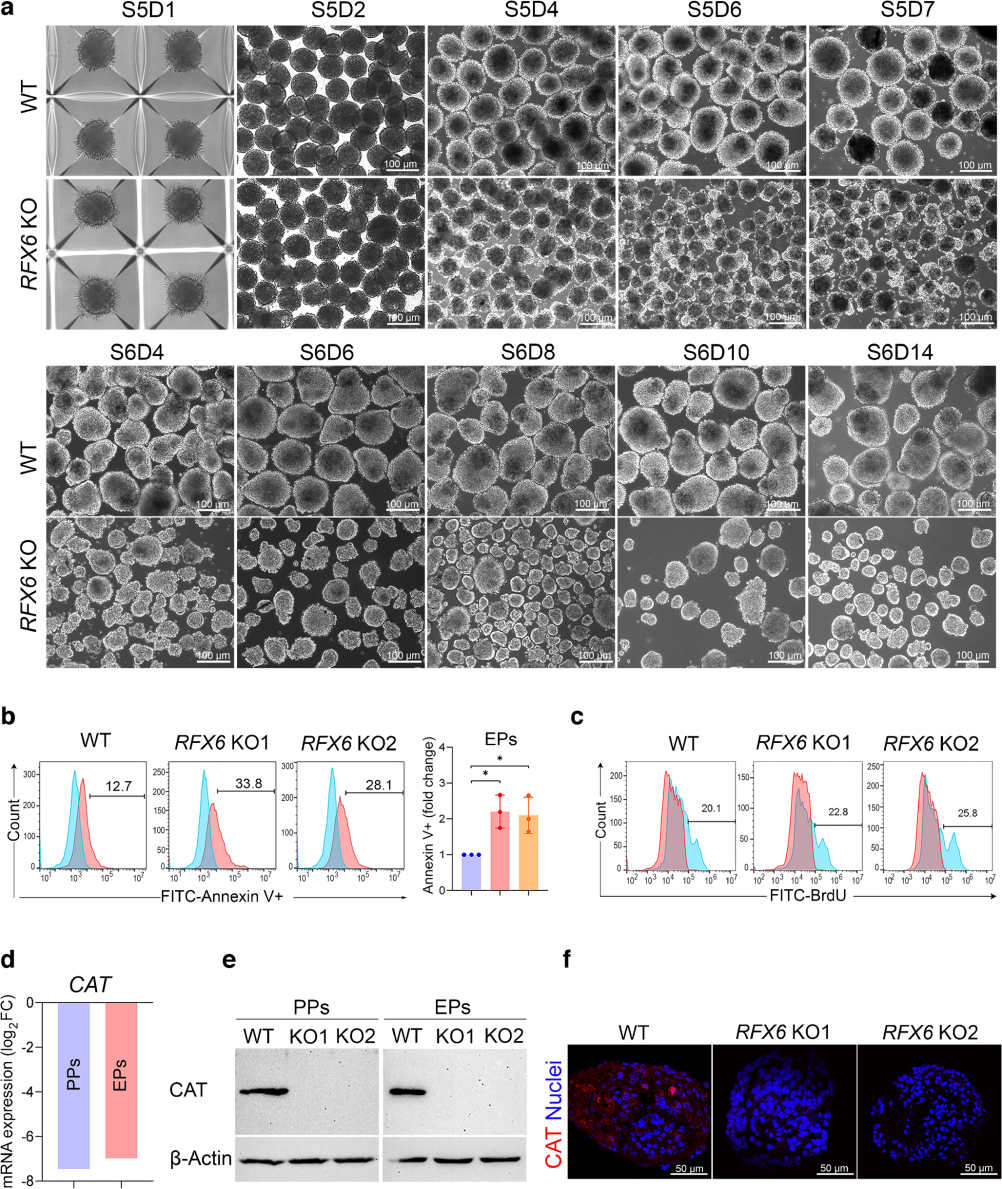
Influence of *RFX6* deletion on pancreatic islet organoid formation and cell viability. (**a**) Comparative morphological analysis of pancreatic islet organoids derived from two *RFX6* KO iPSC lines vs WT iPSCs during differentiation stages 5 and 6 (*n*=3); S, stage, D, day. (**b**) Representative flow cytometry analysis and quantification of apoptosis (Annexin V^+^ cells) on day 3 of stage 5 of differentiation indicates a significant increase in apoptosis in *RFX6* KO EPs in comparison with WT EPs (*n*=3). (**c**) Flow cytometry analysis of BrdU incorporation reveals a slight increase in cell proliferation (BrdU^+^ cells) in EPs derived from *RFX6* KO iPSC lines compared with those derived from WT iPSCs. (**d**) Log_2_ fold change in the expression of *CAT* mRNA in *RFX6* KO PPs and *RFX6* KO EPs compared with WT controls, based on RNA-seq data analysis. (**e**) Western blot analysis showing the absence of CAT protein in *RFX6* KO PPs and *RFX6* KO EPs compared with WT controls. (**f**) Immunofluorescence images showing the lack of CAT expression in *RFX6* KO EPs compared with WT EPs. The data are presented as mean ± SD. *p<0.05; (**d**) PPs *p*=9.62 × 10^−35^; EPs *p*=1.87 × 10^−26^. Scale bar, 50 µm (**f**) or 100 µm (**a**)

To enhance islet differentiation after stage 4, cells were cultured in suspension to form organoids, with an equal number of WT PP and KO PP cells used. Although during the first 2 days of stage 5 no notable difference between WT and KO organoids was observed, a significant variation in organoid size became evident as differentiation progressed. Islet organoids derived from *RFX6* KO iPSCs showed smaller size and irregular shapes compared with those derived from WT iPSCs during stages 5 and 6 (Fig. 5a and ESM Fig. 6a).

To investigate whether the dramatic reduction in islet organoid size could be attributed to either cell death or inhibition of cell proliferation, we conducted apoptosis and proliferation assays during stage 5 of differentiation. Flow cytometry analysis demonstrated a significant increase in the proportion of Annexin V^+^ cells in *RFX6* KO EPs compared with WT EPs (Fig. 5b). Although increased apoptosis was also observed in the final stage of differentiation (stage 6), its level was lower compared with EPs (ESM Fig. 6b), indicating increased cell death with its peak during the EP stage. Quantification of BrdU incorporation revealed no significant difference in proliferation rates between WT and KO cells (Fig. 5c), suggesting that reduced islet organoid size due to *RFX6* loss mainly results from increased cell death.

To elucidate the mechanism underlying the increased cell death, we analysed the top DEGs identified from our RNA-seq data. Interestingly, we observed a significant downregulation of *CAT* (encoding for catalase [CAT], an antioxidant enzyme that is known to protect cells against oxidative stress [31]) in both *RFX6* KO PPs (log_2_ fold change = −7.459; *p*=9.62 × 10^−35^) and *RFX6* KO EPs (log_2_ fold change = −6.978; *p*=1.87 × 10^−26^) compared with WT controls (Fig. 5d). This finding was validated at the protein level through western blot and immunostaining analyses, revealing an almost complete absence of CAT expression in both *RFX6* KO PPs and *RFX6* KO EPs compared with their respective controls (Fig. 5e, f). To validate the role of the CAT in promoting cell survival, we employed the STRING tool for protein functional interaction prediction [27]. Our analysis revealed CAT’s strong interaction with antioxidative stress proteins, such as superoxide dismutase proteins (ESM Fig. 6c).

### *RFX6* loss hinders the development of pancreatic islet cells

**Fig. 6:**
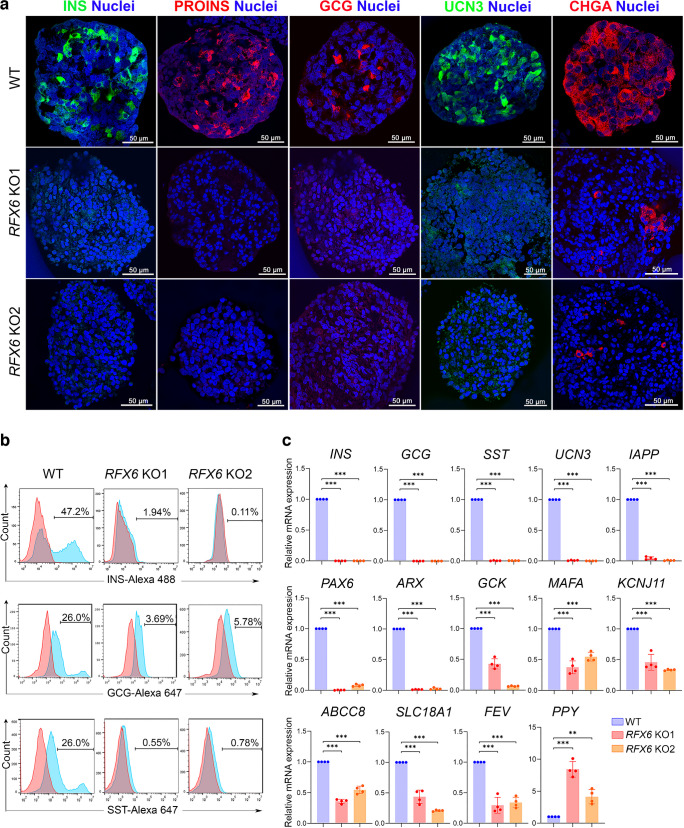
*RFX6* loss impairs the development of pancreatic islet cells. (**a**) Confocal immunofluorescence showing expression of pancreatic islet markers INS, proinsulin (PROINS), GCG, UCN3 and CHGA in islets derived from two different *RFX6* KO iPSC lines compared with WT controls (*n*=3). (**b**) Flow cytometry analysis of the expression of INS, GCG and SST in islets derived from *RFX6* KO iPSCs compared with expression in islets derived from WT iPSCs (*n*=3). (**c**) RT-qPCR analysis for the mRNA expression of key islet genes *INS*, *GCG*, *SST*, *UCN3*, *IAPP*, *PAX6*, *ARX*, *GCK*, *MAFA*, *KCNJ11*, *ABCC8*, *SCL18A1*, *FEV* and *PPY* (*n*=4). Data are represented as mean ± SD; **p<0.01, ***p<0.001. Scale bar, 50 µm

Subsequent differentiation into pancreatic islets demonstrated a lack of expression for INS, proinsulin, GCG, SST and urocortin 3 (UCN3), alongside a significant decrease in CHGA in *RFX6* KO islets compared with WT islets (Fig. 6a, b). This indicates that *RFX6* is essential for the formation of alpha, beta and delta cells. These reductions were confirmed at the mRNA level for *INS*, *GCG*, *SST *and *UCN3* (Fig. 6c). Furthermore, other key pancreatic islet markers, including *IAPP*, *PAX6*, *ARX*, *GCK*, *MAFA*, *KCNJ11*, *ABCC8*, *SLC18A1* and *FEV*, were significantly downregulated (Fig. 6c). On the other hand, pancreatic polypeptide Y (PPY) was significantly upregulated at mRNA and protein levels (Fig. 6c and ESM Fig. 6d). In response to various glucose concentrations, *RFX6* KO islets exhibited no significant changes in INS secretion, with their total INS content being significantly lower than that in WT controls (ESM Fig. 6e).

### RFX6 overexpression rescues the expression of dysregulated genes in pancreatic cells lacking *RFX6*

**Fig. 7:**
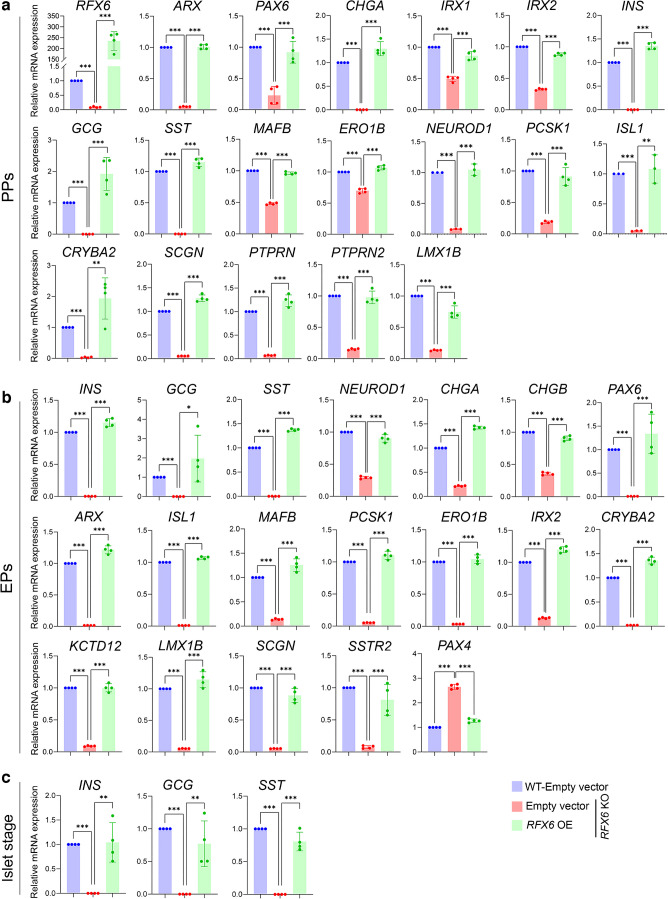
RFX6 overexpression rescues the expression of dysregulated genes in pancreatic cells lacking *RFX6*. (**a**) RT-qPCR analysis for the expression of pancreatic endocrine genes: *RFX6*, *ARX*, *PAX6*, *CHGA*, *IRX1*, *IRX2*, *INS*, *GCG*, *SST*, *MAFB*, *ERO1B*, *NEUROD1*, *PCSK1*, *ISL1*, *CRYBA2*, *SCGN*, *PTPRN*, *PTPRN2* and *LMX1B* in PPs derived from *RFX6* KO iPSCs and WT iPSCs, 48 h following ectopic expression of RFX6 (*n*=4). (**b**) RT-qPCR analysis for the expression of pancreatic endocrine genes: *INS*, *GCG*, *SST*, *NEUROD1*, *CHGA*, *CHGB*, *PAX6*, *ARX*, *ISL1*, *MAFB*, *PCSK1*, *ERO1B*, *IRX2*, *CRYBA2*, *KCTD12*, *LMX1B*, *SCGN*, *SSTR2* and *PAX4*, in EPs derived from *RFX6* KO iPSCs and WT iPSCs, 72 h following ectopic expression of RFX6 (*n*=4). (**c**) RT-qPCR analysis for the expression of *INS*, *GCG* and *SST* in islet organoids at stage 6 following overexpression of RFX6 at the end of stage 4 (*n*=4). Data are represented as mean ± SD; *p<0.05, **p<0.01, ***p<0.001

Next, we aimed to reverse RFX6-associated defects by ectopically expressing *RFX6* (*RFX6* overexpression [OE]). *RFX6* was overexpressed on days 2 and 4 of stage 4 for assessing its effect on PPs and EPs, and subsequently on islets (Fig. 7). At the end of stage 4, the *RFX6* OE significantly increased mRNA expression levels of pancreatic endocrine genes that were downregulated in *RFX6* KO PPs, including *RFX6*, *ARX*, *PAX6*, *CHGA*, *IRX1*, *IRX2*, *INS*, *GCG*, *SST*, *MAFB*, *ERO1B*, *NEUROD1*, *PCSK1*, *ISL1*, *CRYBA2*, *SCGN*, *PTPRN*, *PTPRN2* and *LMX1B* (Fig. 7a). Furthermore, we assessed the impact of *RFX6* OE on the dysregulated DEGs on day 3 of stage 5, 72 h post-transfection. Our results revealed a substantial increase in the expression levels of *INS*, *GCG*, *SST*, *NEUROD1*, *CHGA*, *CHGB*, *PAX6*, *ARX*, *ISL1*, *MAFB*, *PCSK1*, *ERO1B*, *IRX2*, *CRYBA2*, *KCTD12*, *LMX1B*, *SCGN* and *SSTR2* following *RFX6* OE (Fig. 7b). Moreover, it induced a significant decrease in the *PAX4* mRNA levels, which had been upregulated in *RFX6* KO EPs (Fig. 7b). In addition, *RFX6* OE at the end of stage 4 increased the expression of INS, GCG and SST in the *RFX6* KO islets (Fig. 7c).

## Discussion

Recent studies have highlighted the pivotal role of RFX6 in human pancreatic islet development and function, and its association with diabetes [3, 28, 32, 33]. Nonetheless, its exact role in diabetes pathogenesis is still poorly understood and a comprehensive understanding of its specific function during human pancreatic islet development is needed. In this study, we precisely examined RFX6 expression across different stages of hPSC-derived pancreatic islets using different approaches. Furthermore, we developed an isogenic KO platform using human iPSC-derived islets to investigate molecular and cellular alterations at different developmental stages carrying *RFX6* loss-of-function mutations. Our findings are consistent with previous studies, showing the following results: (1) the absence of INS-, GCG- and SST-producing cells and an increase in PPY cell production due to *RFX6* loss; and (2) significant downregulation of genes related to pancreatic endocrine differentiation, INS secretion and ion transport in association with *RFX6* loss [4, 32, 33]. In addition, our study unveils novel insights into the role of RFX6 during pancreatic islet development. Our data indicate the absence of RFX6 does not impede iPSC differentiation into PPs co-expressing PDX1 and NKX6.1, which serve as precursors to pancreatic beta cells. Furthermore, RFX6 deficiency results in the formation of smaller-sized (hypoplastic) islet organoids, potentially driven by increased cellular apoptosis and likely linked to the deficiency of the antioxidant enzyme CAT. These findings imply that pancreatic hypoplasia and the absence of islet cells due to *RFX6* loss-of-function mutations are associated with cellular apoptosis, reduced CAT enzyme expression and reduced pancreatic endocrine gene expression.

Our findings revealed a significant decrease in PDX1 and CDX2 expression in *RFX6* KO PF compared with WT PF, consistent with recent findings [33, 34]. However, the absence of RFX6 did not impact the co-expression of PDX1 and NKX6.1 in PPs. These results align with our timeline expression analysis, which demonstrated the co-localisation of RFX6 with PDX1 in the PF stage, while RFX6 showed no co-expression with PDX1 and NKX6.1 in PPs. The difference in the impact on PDX1 expression between PF and PP stages observed in this study may be attributed to RFX6’s involvement during early differentiation stages in intestinal development, as recently reported in iPSC-derived intestinal models [35]. RFX6 plays a crucial role in both pancreas and small-intestine development, as these organs share a common origin in the gut endoderm [34]. PDX1, crucial for pancreas development, also influences small-intestine development and function. Previous studies suggest that PDX1 acts downstream of RFX6 during gut-tube patterning, with co-expression in EECs of the duodenum and iPSC-derived gut endoderm [35–37]. *RFX6* mutant iPSCs generated defective intestinal organoids due to suppression of PDX1 expression [35]. Our results contradict those of two prior studies. One demonstrated a significant decrease in PDX1 and NKX6.1 levels in PPs derived from MRS patient-specific iPSCs and *RFX6* KO iPSCs [28]. The other study, utilising *RFX6* KO-hESCs, indicated a reduction in the number of PPs due to a marked decrease in PDX1 expression [32]. Our findings suggest that inhibition of PDX1 expression associated with *RFX6* loss prior to the PP stage may disrupt intestinal development, supported by a significant reduction in CDX2 expression, crucial for intestinal development [38]. Furthermore, RFX6 is not essential for forming PDX1^+^/NKX6.1^+^ PPs during pancreatic islet development.

The deficiency of RFX6 led to impaired expression of critical transcription factors and genes essential for endocrine cell development across various stages, including *PAX6*, *INSM1*, *ARX*, *NEUROD1*, *ISL1*, *IRX1*, *IRX2*, *MAFB*, *TTR*, *FEV* and *CHGA* among others. Conversely, the expression of transcription factors such as PDX1, NKX6.1, SOX9 and FOXA2, specific to PPs [39], remained unaffected by the absence of RFX6 in the PPs, while endocrine transcription factors such as paired box 4 (PAX4), NEUROG3 and NKX2.2 were increased in the EPs due to RFX6 deficiency. These findings are consistent with recent results indicating that *RFX6* loss does not affect SOX9 expression and increases the expression of NEUROG3, PAX4 and NKX2.2 [33]. RFX6 acts downstream of NEUROG3 during pancreatic development [1], regulating PAX4 expression [40]. Our re-analysis of single-cell data obtained from different stages of hESC differentiation into pancreatic islets [25] confirmed the highest *RFX6* expression levels in endocrine clusters, including *NEUROG3*^high^/*GHRL*^high^, CHGA^high^/*NEUROD1*^high^, *ERO1LB*^high^/*ARX*^high^, *POU2F2*^high^/*RFX3*^high^, *TTR*^high^/*GCG*^high^, *GCG*^high^/*CHGA*^high^ and *HHEX*^high^/*SST*^high^. The analysis revealed that clusters with high PDX1 and SOX9 expression during the progenitor stages (D11 and D14) did not exhibit RFX6 expression. A recent report highlighted a developmental trajectory emerging at stage 4 (PPs), leading to the formation of primary endocrine cell groups. The differentiation process becomes notably intricate during stage 5 (EPs), primarily due to the presence of numerous subpopulations [40]. A recent study emphasised RFX6’s role in alpha cell function, revealing that its absence leads to impaired exocytosis and GCG secretion, complementing previous findings on beta cell development [41]. These findings underscore the crucial role of RFX6 in regulating pancreatic endocrine genes important for islet cell development, including *GCG* (alpha cells), *INS* (beta cells) and *SST* (delta cells).

Biallelic mutations in *RFX6* are associated with permanent neonatal diabetes mellitus (PNDM), with affected individuals exhibiting smaller size pancreas compared with healthy control individuals [4]. The cause of this pancreatic hypoplasia remains unclear. Recent human studies have suggested that reduced pancreas size may result from suppressed PDX1 expression at the PP stage [28, 32]. However, our current study demonstrated *RFX6* deletion reduced PDX1 in PF without affecting its expression at the PP stage, suggesting other mechanisms. Islet organoids derived from *RFX6* KO iPSCs were smaller in size compared with WT controls due to increased apoptosis during endocrine specification stages. This contradicts a previous *RFX6* KO hESC study suggesting reduced pancreas size is not caused by reduced proliferation or increased apoptosis but from the reduction in PDX1 at early stages of pancreatic development [32]. In our endeavour to unravel the mechanism behind increased cell death in pancreatic cells lacking *RFX6*, our RNA-seq analysis identified antioxidant enzyme CAT downregulation as a potential cause for the increased cell death. Western blotting and immunostaining confirmed the absence of CAT in *RFX6* KO PPs and *RFX6* KO EPs vs WT control PPs and EPs. CAT regulates cellular hydrogen peroxide levels, safeguarding beta cells against oxidative damage [42, 43]. Elevated hydrogen peroxide levels can harm pancreatic beta cells and disrupt INS production [42, 44]. Mutations in *CAT*, elevating hydrogen peroxide, may increase type 2 diabetes risk due to peroxide-induced beta cell damage [45]. Taken together, these findings indicate that RFX6 plays a crucial role in safeguarding pancreatic islets during development by maintaining CAT expression, thereby offering protection against oxidative damage.

In summary, our study explored the effects of *RFX6* deletion on pancreatic islet development. It showed a substantial reduction in PDX1 expression in *RFX6* KO PF, consistent with earlier studies. However, the absence of RFX6 did not disrupt the development of PDX1 and NKX6.1 in PPs, aligning with the lack of RFX6 co-expression with key progenitor markers and its absence in cell clusters expressing high levels of PDX1 and SOX9 in PPs. RFX6’s role in early intestinal development may explain the PDX1 expression differences between PFs and PPs. Furthermore, our findings indicate that RFX6 regulates the expression of crucial pancreatic endocrine genes essential for the formation of INS-, GCG- and SST-expressing cells during pancreatic differentiation. Moreover, *RFX6* deletion resulted in smaller islet organoids, attributed to increased cell apoptosis during endocrine specification. These results underscore RFX6’s pivotal role in safeguarding pancreatic islets, potentially explaining pancreatic hypoplasia in individuals with *RFX6* homozygous mutations. Thus, our study highlights the complexity of RFX6’s role in pancreatic islet development and its implications for understanding pancreatic hypoplasia and diabetes risk.

## Supplementary Information

Below is the link to the electronic supplementary material.

Supplementary file1 (PDF 10398 KB)

## Data Availability

RNA-seq datasets have been deposited in the Zenodo repository with accession link (DOI: 10.5281/zenodo.10656891).

## Abbreviations

CAT: Catalase
CDX2: Caudal type homeobox 2
CHGA: Chromogranin A
DEG: Differentially expressed gene
EEC: Enteroendocrine cell
EP: Endocrine progenitor
FOXA2: Forkhead box A2
GCG: Glucagon
GIP: Gastric inhibitory polypeptide
GO: Gene ontology
HA: Haemagglutinin
hESC: Human embryonic stem cell
INS: Insulin
iPSC: Induced pluripotent stem cell
KO: Knockout
MRS: Mitchell–Riley syndrome
NEUROG3: Neurogenin 3
NKX2.2: NK2 homeobox 2
NKX6.1: NK homeobox 1
PAX4: Paired box 4
PDX1: Pancreatic and duodenal homeobox 1
PF: Posterior foregut
PNDM: Permanent neonatal diabetes
PP: Pancreatic progenitor
PPY: Pancreatic polypeptide Y
RFX6: Regulatory factor X6
*RFX6* OE: RFX6 overexpression
scRNA-seq: Single-cell RNA-seq
SOX: SRY-box transcription factor
SST: Somatostatin
UCN3: Urocortin 3
WT: Wild-type
