# Supplementary material for: Deletion of RFX6 impairs iPSC-derived islet organoid development and survival, with no impact on PDX1+/NKX6.1+ progenitors
Source: Diabetologia. 2024 Jul 30;67(12):2786–803. doi: 10.1007/s00125-024-06232-2 (PMC11604831; doi:10.1007/s00125-024-06232-2)
Supplement: Supplementary file 1 — Supplementary file1 (PDF 10398 KB) [file 125_2024_6232_MOESM1_ESM.pdf]

Electronic Supplementary Material (ESM)

ESM Methods

Differentiation of hESCs and iPSCs into pancreatic islets

hESC/iPSC lines were differentiated *in vitro* into PPs using our protocol [1]. Veres et al. protocol was adapted for further differentiation into pancreatic islet cells [2]. iPSCs underwent 2D culture until the PP stage, then dissociated into single cells using TryplE. Cells were counted and seeded into Aggrewell 400 24-well plates (Stem Cell Technologies, Vancouver, Canada) to form organoids. Each well received 2x10^6 cells (~1,666 cells per microwell). After 48 hours, organoids were transferred to ultra-low attachment plates for continued differentiation on a shaker. ESM Table 1 details the differentiation reagents used.

Paraffin embedding and immunofluorescence

For cultured cells, immunostaining was conducted on cells that had been fixed using 4% paraformaldehyde (PFA) in PBS for 20-25 min, following a protocol described earlier [3]. Briefly, cells were permeabilized with PBS containing 0.5% Triton (PBST) for 15 minutes and were blocked overnight at 4°C using 6% bovine serum albumin (BSA) in PBST. Primary antibodies were added overnight at 4°C then cells were washed three times using TBST containing 0.5% Tween (TBST). Secondary antibodies were added at room temperature for 1 hour followed by three washes with TBST. Nuclear staining was done using Hoechst 33258 diluted 1:5000 in PBS (Life Technologies, California, USA). Cells were washed using PBS and imaged using inverted

fluorescence microscope (Olympus, Tokyo, Japan). Used antibodies' details are listed in ESM Table 2.

For the 3D pancreatic islet organoids, we employed the paraffin embedding technique. Islet organoids were collected into a 1.5 mL Eppendorf tube, washed with PBS, and subsequently fixed with 4% PFA for 20-30 minutes on a shaker at room temperature. After fixation, the organoids underwent two washes with TBST followed by the addition of PBS The organoids were then mixed with 100 μL of melted Histogel (Epredia, New Hampshire, USA) in a 7x7x5 mm disposable embedding mold. These histogel blocks were released from the mold and wrapped in a 3-ply tissue and inserted into tissue cassettes. The dehydration series for the blocks was adapted from Kong et al.'s paraffin embedding protocol [4]. The paraffin blocks were then sectioned using a microtome at a thickness of 5 μm. Antigen retrieval was performed using the method adapted from Campbell-Thompson et al.'s [5]. Subsequently, slides were permeabilized using PBST for 15 minutes at room temperature and blocked with 6% BSA in PBST for at least 2 hours at room temperature. The subsequent steps followed were similar to those in immunofluorescence.

Flow cytometry

Differentiated cells were collected at different stages from one well of a 6-well plate. Cells were first washed once with PBS and then dissociated using TrypLE into single cells. Afterwards, cells were collected in a 15 mL Falcon Tube (ThermoFisher Scientific, Massachusetts, USA) and resuspended in 1 mL of PBS. All cell centrifugations were done at 1800 rpm for 5 minutes (Eppendorf, Enfield, USA). Cells were resuspended in 200 μL of cold PBS and then 1 mL of 4% PFA was added then incubated, slanted, at room temperature for 20 minutes on a shaker. Cells were washed twice with TBST then permeabilized using PBST. Cells were blocked using 6% BSA

in PBST overnight at 4°C. 1-2x10^5 of cells, around 100 µL, were transferred into V-bottom shaped 96-well plate (Corning, New York, USA) and primary antibodies were added for 3 hours on a shaker, at room temperature. Cells were washed twice with TBST then incubated with secondary antibodies diluted in PBS for 30 minutes at room temperature. Cells were washed again twice and resuspended in PBS then run using BD Accuri C6 Flow Cytometer then analysed using FlowJo software. Used antibodies' details are listed in ESM Table 2.

Western blotting

Total protein was extracted from 1-2 wells of a 6-well plate using RIPA lysis buffer with protease inhibitor (ThermoFisher Scientific, Massachusetts, USA). Protein concentration was measured using Pierce BCA kit (ThermoFisher Scientific, #23225, Massachusetts, USA). 20-30 µg of total protein were loaded and separated using 7.5-10% SDS-PAGE gels then transferred onto PVDF membranes (ThermoFisher Scientific, #88518, Massachusetts, USA). Membranes were blocked using 15% skimmed milk in TBST at least for 3 hours at room temperature or overnight at 4°C. Primary antibody was then added and incubated overnight at 4°C. Membranes were then washed using TBST and secondary antibody was added for 1 hour at room temperature followed by more TBST washes. Membranes were developed using SuperSignal West Pico Chemiluminescent substrate (ThermoFisher Scientific, #34580, Massachusetts, USA). Used antibodies' details are listed in ESM Table 2.

RNA extraction, PCR, and RT-qPCR

Cells were collected from one well of a 6-well plate using 700 µL of TRIzol Reagent (Life Technologies, California, USA) and RNA was extracted using Direct-zol RNA Miniprep (Zymo Research, California, USA). 1µg of total RNA was used for cDNA synthesis using High-Capacity cDNA Reverse Transcription Kit while following manufacturer's protocol (Applied Biosystems). PCR was done using 2X PCR Master Mix (ThermoFisher Scientific, #K0171, Massachusetts, USA). RT-qPCR was performed using GoTaq qPCR SYBR Green Master Mix (Promega, Madison, USA). GAPDH was used as an endogenous control (primer details are listed in ESM Table 3).

Single cell analysis

The online published GSE202497 data set (https://www.ncbi.nlm.nih.gov/geo/query/acc.cgi?acc=GSE202497) was used [10] and re-analyzed. In total, 25686 cells were retrieved from the dataset. The data consists of 5 samples from human pluripotent stem cell differentiation into islets at D11, D14, D21, D32, D39 days. Top 2000 highly variable genes were obtained using Seurat V3 algorithm implement in Scanpy. The data was log-normalized and Harmony with "sample" as batch variable was used for technical batch-effect correction. Neighborhood graph was computed using the sc.pp.neighbors function using top 50 batch-effect adjusted principal components. Clustering at different resolutions was performed using the leiden algorithm. Marker genes for each cluster were calculated using the Wilcoxon method implemented in rank_genes_groups function. Finally, using the marker genes, the clusters were manually annotated. Top marker genes and cluster names are provided in ESM Fig. 2.

RNA-sequencing analysis

The mRNA isolation utilized the NEBNext Poly(A) mRNA Magnetic Isolation Kit (New England Biolabs, #E7490, Massachusetts, USA) with 1 μg of total RNA. Subsequently, RNA-seq libraries were generated using the NEBNext Ultra Directional RNA Library Prep Kit (New England Biolabs, #E7420L, Massachusetts, USA), followed by sequencing on an Illumina Hiseq 4000 system. Raw data underwent conversion to FASTQ files through Illumina BCL2Fastq Conversion Software v2.20. For intial preprocessing of the Pair-end FASTQ files, nf-core/rnaseq (version 2.7.2) pipeline implemented in the nextflow workflow (version 23.10.1) was used. STAR (version 2.7.9a) was used for the read alignments, Salmon (version 1.5.0) for quantification of reads, TrimGalore (version 0.6.6) was applied for read trimming and GENCODE (version 38) was used for annotation of genes [7]. The count matrix generated by Salmon was filtered, excluding genes labeled 'Mt_tRNA,' Mt_rRNA,'rRNA' and 'rRNA_pseudogene' in the GENCODE annotation file. Subsequently, low expression genes were excluded using the HTSFilter (version 1.32.0) [8]. Differential expression analysis was performed using the DESeq2 (version 1.32.0) to identify the DEGs for specific comparisons. PCA was generated using the glmpca package of R. DEGs were identified based on criteria of log2 fold change (FC) > 1 and <-1, with a P-value < 0.05. Subsequent analyses also included Gene Ontology (GO) and Kyoto Encyclopaedia of Genes and Genomes (KEGG) pathways, performed using the Database for Annotation, Visualization, and Integrated Discovery (DAVID) [9].

Apoptosis and proliferation assays

The apoptosis assay was performed using the Annexin V-FITC Apoptosis Detection Kit (Abcam, #ab14085, Cambridge, UK), while the proliferation assay was conducted employing the BrdU

incorporation method (ThermoFisher Scientific, #000103, Massachusetts, USA), following previously established protocols [6]. For the proliferation assay, cells were exposed to 20 μM BrdU in differentiation media for 5-6 hours at 37°C. Subsequently, the cells were washed with PBS, dissociated using TryplE, and fixed with cold 70% ethanol overnight at 4°C. Following this, the cells were rinsed once with PBS and denatured with 0.2 M HCL containing 0.5% Triton for 15-20 minutes at room temperature, followed by a 0.1 M sodium tetraborate treatment for another 15-20 minutes. Afterward, the cells were washed with PBS and blocked using 6% BSA in PBS with 0.1% saponin. Cells were then incubated with Alexa Fluor 488-conjugated BrdU monoclonal antibody (ThermoFisher Scientific, #B35130, 1:100, Massachusetts, USA) overnight at 4°C. Cells were then washed with TBST twice and quantification of BrdU-positive cells was conducted through flow cytometry analysis using BD Accuri C6 Flow Cytometer.

RFX6 overexpression

Single cells were transfected with either RFX6 plasmid (RFX6 (Myc-DDK-tagged), RC206174, OriGene, USA) or an empty vector using Lipofectamine 3000 according to the manufacturer's protocol (ThermoFisher Scientific, #L3000-015, Massachusetts, USA). For stage 4, cells were transfected at the end of day 2 of stage 4 and collected 48 hours later (day 4 of stage 4). For stage 5, cells were transfected at the end of stage 4 and collected 72 hours later (day 3 of stage 5). For stage 6, cells were transfected at the end of stage 4 and collected on day 7 of stage 6.

ESM References

[1] Memon B, Karam M, Al-Khawaga S, Abdelalim EM (2018) Enhanced differentiation of human pluripotent stem cells into pancreatic progenitors co-expressing PDX1 and NKX6.1. Stem Cell Res Ther 9(1): 15. 10.1186/s13287-017-0759-z

[2] Veres A, Faust AL, Bushnell HL, et al. (2019) Charting cellular identity during human in vitro beta-cell differentiation. Nature 569(7756): 368-373. 10.1038/s41586-019-1168-5

[3] Elsayed AK, Younis I, Ali G, Hussain K, Abdelalim EM (2021) Aberrant development of pancreatic beta cells derived from human iPSCs with FOXA2 deficiency. Cell Death Dis 12(1): 103. 10.1038/s41419-021-03390-8

[4] Kong Y, Ebrahimpour P, Liu Y, Yang C, Alonso LC (2018) Pancreatic Islet Embedding for Paraffin Sections. Journal of visualized experiments : JoVE(136). 10.3791/57931

[5] Campbell-Thompson ML, Heiple T, Montgomery E, Zhang L, Schneider L (2012) Staining protocols for human pancreatic islets. Journal of visualized experiments : JoVE(63): e4068. 10.3791/4068

[6] Aghadi M, Elgendy R, Abdelalim EM (2022) Loss of FOXA2 induces ER stress and hepatic steatosis and alters developmental gene expression in human iPSC-derived hepatocytes. Cell Death Dis 13(8): 713. 10.1038/s41419-022-05158-0

[7] Patro R, Duggal G, Love MI, Irizarry RA, Kingsford C (2017) Salmon provides fast and bias-aware quantification of transcript expression. Nat Methods 14(4): 417-419. 10.1038/nmeth.4197

[8] Rau A, Gallopin M, Celeux G, Jaffrezic F (2013) Data-based filtering for replicated high-throughput transcriptome sequencing experiments. Bioinformatics 29(17): 2146-2152. 10.1093/bioinformatics/btt350

[9] Huang DW, Sherman BT, Lempicki RA (2009) Systematic and integrative analysis of large gene lists using DAVID bioinformatics resources. Nature Protocols 4(1): 44-57. 10.1038/nprot.2008.211

[10] Zhu H, Wang G, Nguyen-Ngoc KV, et al. (2023) Understanding cell fate acquisition in stem-cell-derived pancreatic islets using single-cell multiome-inferred regulomes. Dev Cell 58(9): 727-743 e711. 10.1016/j.devcel.2023.03.011

ESM Tables

ESM Table 1. *In vitro* pancreatic differentiation protocol.

Stage of Differentiation	Media	Final Cytokine Concentration
Stage 1:<br>Definitive Endoderm<br>(Day 1)	MCDB131<br>1% Pen/Strep<br>1% L-Glutamine<br>10 mM Glucose<br>0.5% BSA<br>1.5 g/L $NaHCO_3$	100 ng/mL Activin A<br>0.25 mM Vitamin C<br>2 µM CHIR99021<br>1 mM Rock Inhibitor
Stage 1:<br>Definitive Endoderm<br>(Days 2-3)	MCDB131<br>1% Pen/Strep<br>1% L-Glutamine<br>10 mM Glucose<br>0.5% BSA<br>1.5 g/L $NaHCO_3$	100 ng/mL Activin A<br>0.25 mM Vitamin C
Stage 2:<br>Primitive Gut Tube<br>(Days 4-5)	MCDB131<br>1% Pen/Strep<br>1% L-Glutamine<br>10 mM Glucose<br>0.5% BSA<br>1.5 g/L $NaHCO_3$	3 ng/mL Wnt3a<br>0.75 µM Dorsomorphin<br>0.25 mM Vitamin C<br>50 ng/mL FGF10
Stage 3:<br>Posterior Foregut<br>(Days 6-7)	DMEM<br>1% Pen/Strep<br>1% L-Glutamine<br>4.5 g/L D-Glucose<br>110 mg/L Sodium Pyruvate	1% B27<br>200 nM LDN<br>0.25 mM Vitamin C<br>50 ng/mL FGF10<br>2 µM Retinoic acid<br>0.25 µM SANT-1
Stage 4:<br>Pancreatic Progenitor<br>(Days 8-11)	DMEM<br>1% Pen/Strep<br>1% L-Glutamine<br>4.5 g/L D-Glucose<br>110 mg/L Sodium Pyruvate	1% B27<br>200 nM LDN<br>100 ng/mL EGF<br>0.25 mM Vitamin C<br>10 mM Nicotinamide
Stage 5:<br>Endocrine<br>Progenitor<br>(Days 12-15)	MCDB131<br>1% Pen/Strep<br>1% L-Glutamine<br>20% BSA<br>1.754 g/L $NaHCO_3$<br>20 mM Glucose<br>5 mL ITS	0.25 µM SANT-1<br>20 ng/mL Beta-Cellulin<br>1 µM Gamma secretase inhibitor<br>10 µM Alk5i<br>1 µM T3<br>0.1 µM Retinoic acid<br>0.25 mM Vitamin C

	10 mg/L Heparin	
Stage 5:<br>Endocrine<br>Progenitor<br>(Days 16-18)	MCDB131<br>1% Pen/Strep<br>1% L-Glutamine<br>20% BSA<br>1.754 g/L $NaHCO_3$<br>20 mM Glucose<br>5 mL ITS<br>10 mg/L Heparin	20 ng/mL Beta-Cellulin<br>1 µM Gamma secretase inhibitor<br>10 µM Alk5i<br>1 µM T3<br>0.1 µM Retinoic acid<br>0.25 mM Vitamin C
Stage 6:<br>Pancreatic Islet<br>(Days 19-32)	MCDB131<br>1% Pen/Strep<br>1% L-Glutamine<br>20% BSA<br>1.23 g/L $NaHCO_3$<br>2.5 mM Glucose<br>5 mL ITS	0.25 mM Vitamin C

ESM Table 2. List of used antibodies for immunostaining and Western blot.

Antibody	Company	Catalog No.	Dilution
B-actin	Santa Cruz	Sc-47778	WB (1:10,000)
CAT	Cell Signaling	12980S	WB (1:4000)<br>IS (1:1000)
CDX2	Abcam	ab76541	WB (1:2500)<br>IS (1:1000)
CHGA	ThermoFisher	MA5-14536	WB (1:4000)<br>IS (1:3000)
FOXA2	Cell Signaling	3143	WB (1:4000)<br>IS (1:1000)
FOXA2	Abcam	ab60721	IS (1:1000)
GATA6	R&D	AF1700	IS (1:500)
GCG	Sigma	G2654	IS (1:2000)<br>FACs (1:100)
HA	Cell Signaling	3724s	IS (1:1000)
INS	DSHB	GN-ID4-s	IS (1:2000)<br>FACs (1:100)
OCT4	Cell Signaling	9656	IS (1:500)
NANOG	Cell Signaling	9656	IS (1:500)
NGN3	R&D	AF3444	IS (1:1000)
NKX2.2	DSHB	74.5A5-c	IS (1:2000)
NKX6.1	DSHB	F55A12-C	WB (1:4000)<br>IS (1:2000)<br>FACs (1:100)
PDX1	Abcam	ab47308	WB (1:4000) / (1:1000)<br>IS (1:500)<br>FACs (1:100)
PROINS	R&D	MAB13361	IS (1:2000)

RFX6	Sigma	HPA037696	WB (1:200)
SOX2	Cell Signaling	9656	IS (1:500)
SOX9	Sigma	HPA001758	WB (1:4000)
SOX17	Origene	CF500096	IS (1:500)
SSEA4	Cell Signaling	9656	IS (1:500)
SST	Millipore	MAB354	FACs (1:100)
TRA-1-60	Cell Signaling	9656	IS (1:500)
TRA-81	Cell Signaling	9656	IS (1:500)
UCN3	Sigma	HPA038281	IS (1:1000)
594 donkey anti-rabbit	Invitrogen	A21207	IS (1:500)
488 donkey anti-rabbit	Invitrogen	A21206	IS (1:500)
568 donkey anti-mouse	Invitrogen	A10037	IS (1:500)
488 donkey anti-mouse	Invitrogen	A21202	IS (1:500)<br>FACs (1:500)
647 donkey anti-mouse	Invitrogen	A31571	FACs (1:500)
568 donkey anti-goat	Invitrogen	A11057	IS (1:500)
488 donkey anti-sheep	Invitrogen	A11015	IS (1:500)
488 goat anti-rat	Invitrogen	A11006	IS (1:500)<br>FACs (1:500)
568 goat anti-rat	Invitrogen	A11077	IS (1:500)
647 donkey anti-rat	Invitrogen	A48272	FACs (1:500)
488 goat anti-guinea pig	Invitrogen	A11073	IS (1:500)

647 goat anti-guinea pig	Invitrogen	A21450	FACs (1:500)
Peroxidase AffiniPure Donkey anti-Rabbit IgG (H+L)	Jackson ImmunoResearch Laboratories	711-035-152	WB (1:10,000)
Peroxidase AffiniPure Donkey anti-Mouse IgG (H+L)	Jackson ImmunoResearch Laboratories	715-035-150	WB (1:10,000)
Peroxidase AffiniPure Donkey anti-guinea pig IgG (H+L)	Jackson ImmunoResearch Laboratories	706-035-148	WB (1:10,000)

ESM Table 3. List of primers used for RT-qPCR validation.

Gene	Forward	Reverse
ABCC8	TTGCCGAAACCGTAGAAGG	CGGTTCCAGCAGAAGCTTC
ARX	CTGCTGAAACGCAAACAGAGGC	CTCGGTCAAGTCCAGCCTCATG
CDX2	CTGGAGCTGGAGAAGGAGTTTC	ATTTTAACCTGCCTCTCAGAGAGC
CHGA	GAAGAAGGCCCCACTGTAGT	TTCCCAGCTCCATCCACAG
CHGB	CACGCCATTCTGAGAAGAGC	TCTCCTGGCTCTTCAAGGTG
CPA1	ACTACGCCACCTACCACACC	GGTGTTGCCAATCTGGATCT
CPA2	ATCTTCCTCCTGCCAGTCAC	CACACCAACACAGAGGCTTC
CRYBA2	GATGTGGGTTCCCTCAAAGT	GCTCACCGTAAGTACAGAACTC
ERO1B	TGAACCCAGAGCGTTACACT	GGCGCCAGAGGATTTAAAGG
FEV	GCCTCTCCAAACTCAACCTC	CAAGCTGGGACTGGGGTAG
FOXA2	GGGAGCGGTGAAGATGGA	TCATGTTGCTCACGGAGGAGTA
GAPDH	ACGACCACTTTGTCAAGCTCATTTC	GCAGTGAGGGTCTCTCTCTTCCTCT
GCG	CTCTTCACCTGCTCTGTTCTAC	TGGATTTCTCCTCTGTGTCTTG
GCK	GCATCTTCCAGCTCTTCGAC	GGGCTACATTTGAAGGCAGA
IAPP	TTGAGAAGCAATGGGCATCC	GGGTGTAGCTTTCAGATGGTTC
INS	AAGAGGCCATCAAGCAGATCA	CAGGAGGCGCATCCACA
INSM1	TTTGTCTCGTGGTTGGAAGC	CCAAAACAACCCGTACGCTA
IRX1	CAAGAATCCCTACCCCACCA	TCCCCATGTCACCTTGTTCT
IRX2	TCACCAAGATGACCCTCACC	TTCGCTTTTGTTTCTCGGGG
ISL1	CTGTGGACATTACTCCCTCTTAC	GCAACCAACACATAGGGAAATC
KCNJ11	GCGCTTTGTGCCCATTGTA	TTGACGGTGTTGCCAAACTTG
KCTD12	GCCTCTGTCACTCAAGTCTTAC	TGACAGGGTGCAGTGAATAAG
LMX1B	ACCTCCTTAACCAGCCTCAG	GCATGGAGTAGAGCCGGTC
MAFA	GCACATTCTGGAGAGCGAGA	CCGCCAGCTTCTCGTATTTC
MAFB	GGAGAATGAGAAGACGCAGC	GTTTCTCGCACTTGACCTTGT
NEUROD1	GCCCCAGGGTTATGAGACTAT	GAGAACTGAGACACTCGTCTGT
NEUROG3	GGCTGTGGGTGCTAAGGGTAAG	CAGGGAGAAGCAGAAGGAACAA
NKX2.2	AAACCATGTCACGCGCTCA	GGCGTTGTACTGCATGTGCT
NKX6.1	GGGCTCGTTTGGCCTATTCGTT	CCACTTGGTCCGGCGGTTCT
ONECUT2	GCCATCTTCAAGGAGAACAAAC	CGTTCATGAAGAAGTTGCTGAC

PAX4	AGCAGAGGCACTGGAGAAAGAGTT	CAGCTGCATTTCCCACTTGAGCTT
PAX6	GCGGAAGCTGCAAAGAAATAG	GGGCAAACACATCTGGATAATG
PCSK1	TCACACATGGGGAGAGAACC	TCCCGTGCAAAATCAGCTTC
PDX1	CGTCCAGCTGCCTTTCCCAT	CCGTGAGATGTACTTGTTGAATAGGA
PPY	AGGTGCTCGCTTGGTCTAGTG	ACCCAGCAGTGGCTGTAGTAAC
PTPRN	CCTACCAAGCAGAGCCAAAC	TGGTCATAGGGCAGGAAGTC
PTPRN2	ACTGAGGATGTGGAGAAGGC	TGAGTTTGCTTTTCGACCCG
RFX6	GTCGATGCATGGCTTGGACT	TGGGCCATAGCTAGACGGTG
SCGN	CTGGGTACTGATGACACGGT	CCAGCAAGCTCTTTCATCCG
SIX3	GCGACTCGGAATGTGATGTAT	GGAGAAGGAAGAGGAGGAAGA
SLC18A1	ATGGTCATCACTGGGGTCAT	GGCTTCTGGGTTGCATACAT
SOX2	GGGAAATGGGAGGGGTGCAAAAGAGG	TTGCGTGAGTGTGGATGGGATTGGTG
SOX9	GACTACACCGACCACCAGAACTCC	GTCTGCGGGATGGAAGGGA
SST	AGCTGCTGTCTGAACCCAAC	CCATAGCCGGGTTTGAGTTA
SSTR2	GCTGTGCCAACCCTATCCTA	TCCTGCTTACTGTCACTCCG
TTR	CATGGGCTCACAACTGAGGA	TTGGCTGTGAATACCACCTCTG
UCN3	GATGGGCTTGGCTTTGTAGA	GGAGGGAAGTCCACTCTCG

ESM Figures

ESM Figure 1

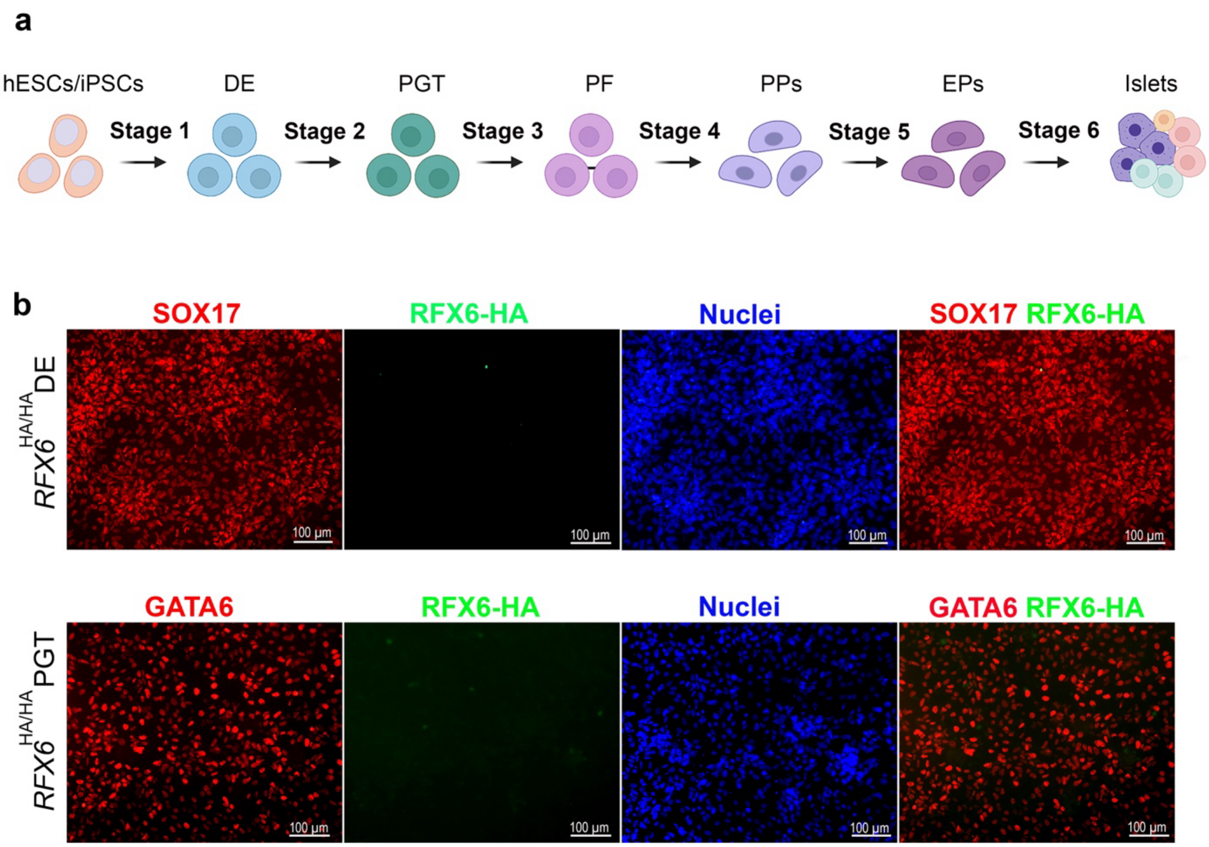

ESM Fig 1. Expression of RFX6 during early stages of hESC differentiation. (A) Diagram illustrating the procedural outline for the differentiation of iPSCs/hESCs into pancreatic islets. (B) Immunostaining showing the expression of RFX6 during differentiation of hESC-H9 into stage 1 (DE) and stage 2 (PGT). DE: definitive endoderm, PGT: primitive gut tube, PF: posterior foregut, PPs: pancreatic progenitors, EPs: endocrine progenitors. Scale bars = 100 μm.

ESM Figure 2

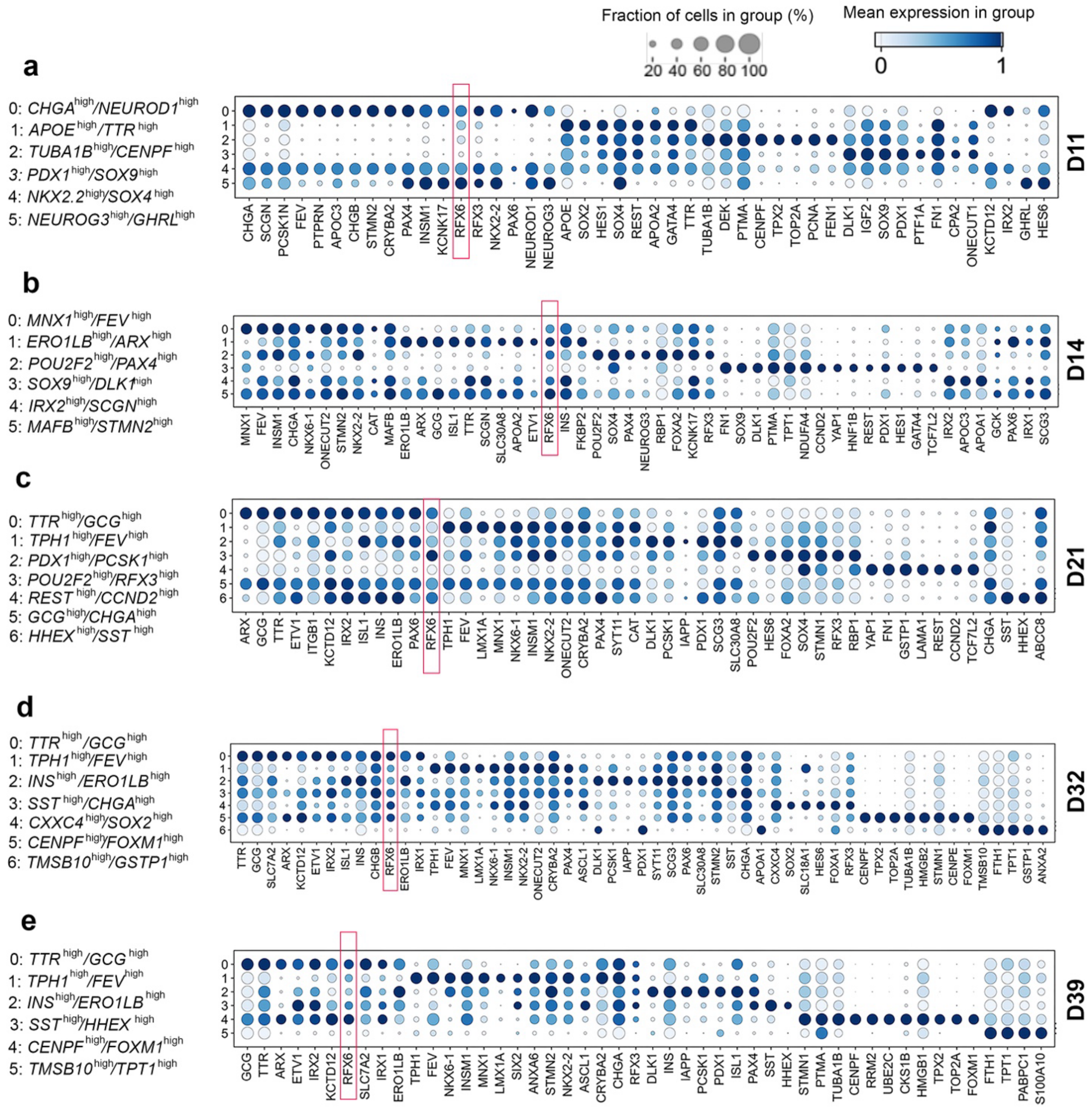

ESM Fig 2. Single-cell sequencing analysis of hESC-derived pancreatic islets. Dot plot showing marker gene expressions across the different cell populations at different days of differentiation, including day 11 (D11) (A), day 14 (D14) (B), day 21 (D21) (C), day 32 (D32) (D), and day 39 (D39) (E).

ESM Figure 3

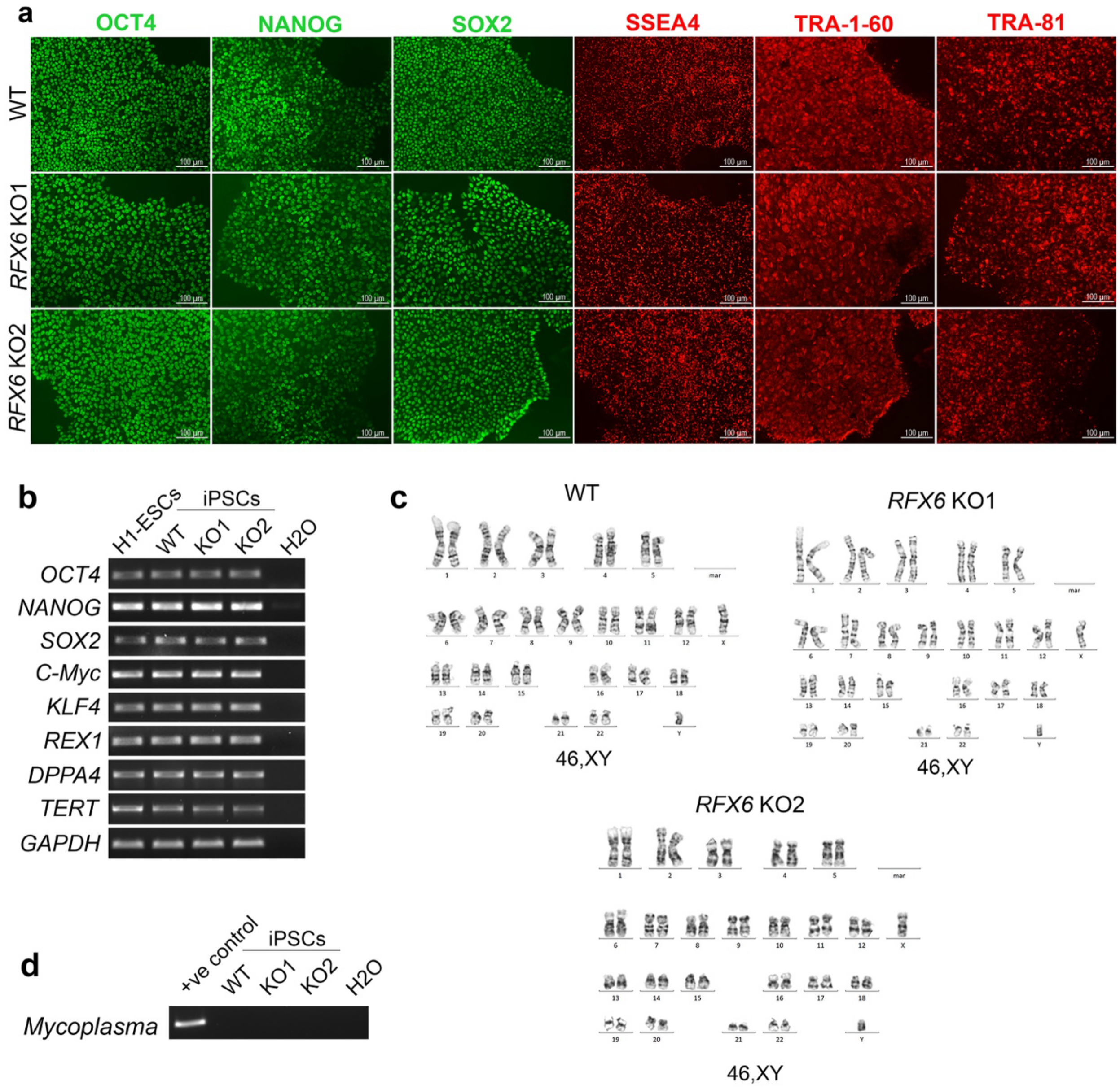

ESM Fig 3. CRISPR/Cas9-edited iPSC lines are pluripotent. (A) Immunofluorescence staining showcases the expression of pluripotency markers OCT4, NANOG, SOX2, SSEA4, and TRA-1-81 in both WT and *RFX6* KO iPSC clones, with nuclei counterstained using Hoechst (blue). (B) RT-PCR analysis depicts the expression of pluripotency-associated genes *OCT4, NANOG, SOX2, C-MYC, KLF4, REX1, DPPA4,* and *TERT*. (C) Karyotype analysis reveals a normal karyotype in both WT and *RFX6* KO iPSC clones. (D) PCR analysis confirms the absence of mycoplasma contamination in the WT and *RFX6* KO iPSC lines. Scale bar = 100 µm.

ESM Figure 4

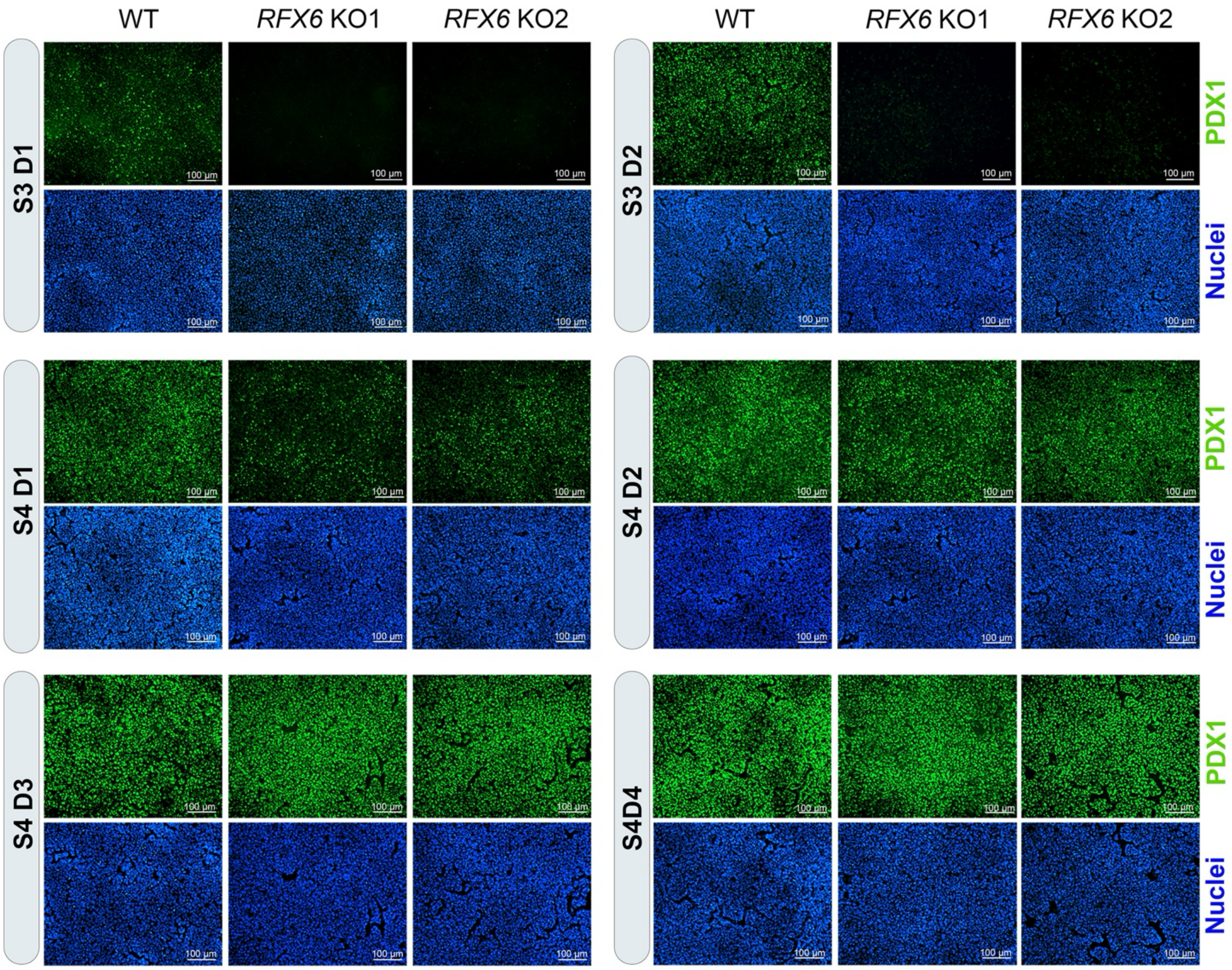

ESM Fig 4. Impact of RFX6 loss on the expression pattern of PDX1 during stages 3 and 4 of differentiation. Immunostaining images showing PDX1 expression on various days, starting from day 1 of stage 3 (S3D1) through stage 4 day 4 (S4D4). Results showed delayed PDX1 expression in *RFX6* KO at PF stage, where the expression initiates from day 1 of stage 4 (PP stage), reaching similar expression to WT at the end of stage 4 day 4. Scale bar = 100 µm.

ESM Figure 5

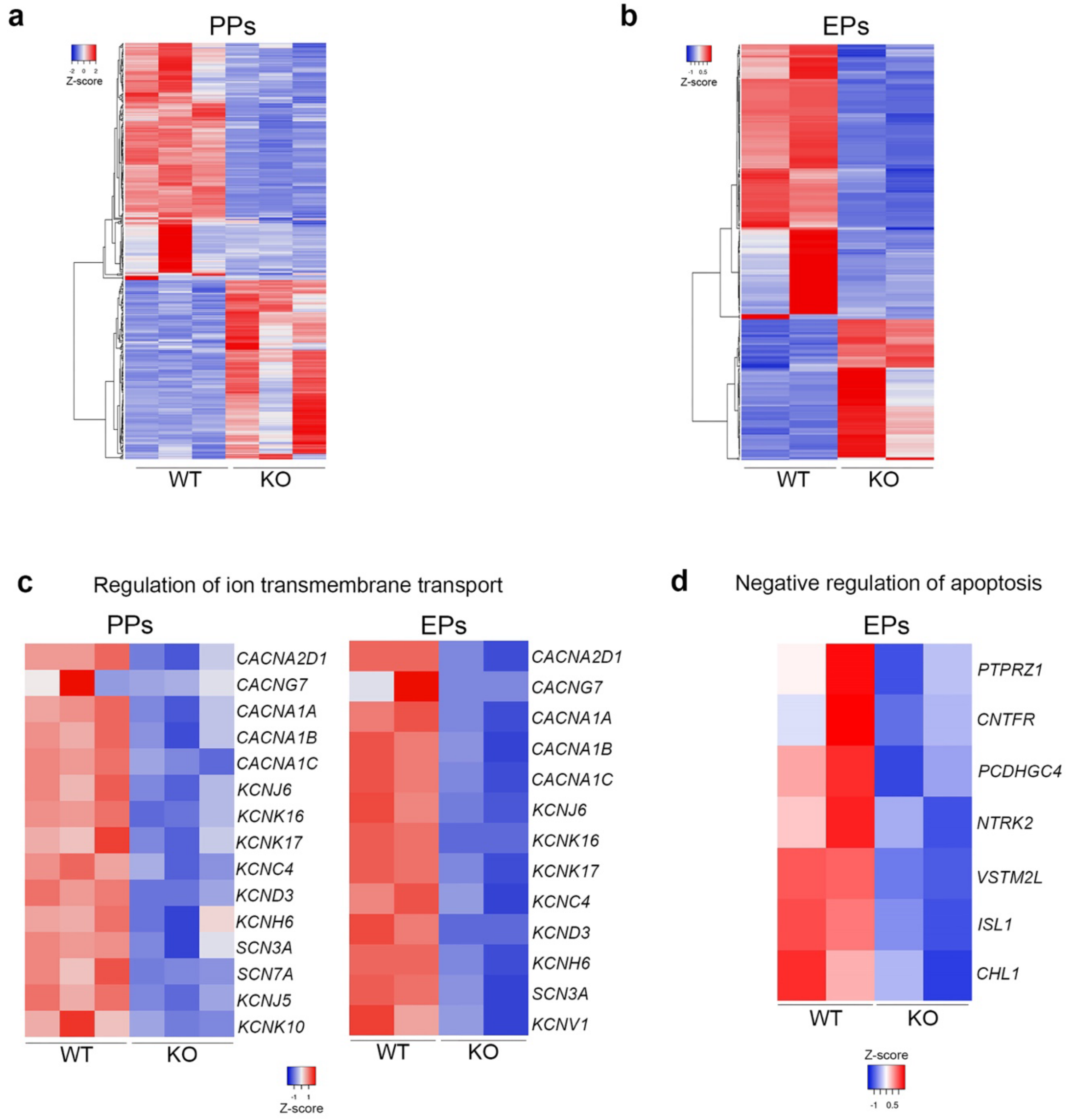

ESM Fig 5. RNA-seq analysis of pancreatic progenitors (PPs) and endocrine progenitors (EPs) derived from WT and *RFX6* KO iPSCs. A clustering heatmap of differentially expressed genes (DEGs) in PPs (A) and EPs (B) derived from WT and *RFX6* KO iPSCs. Heatmaps of DEGs associated with the regulation of ion transmembrane transport in *RFX6* KO PPs and *RFX6* KO-EPs (C), and negative regulation of apoptosis (D) compared to WT controls (p-value <0.05 and Log2 FC< -1.0).

ESM Figure 6

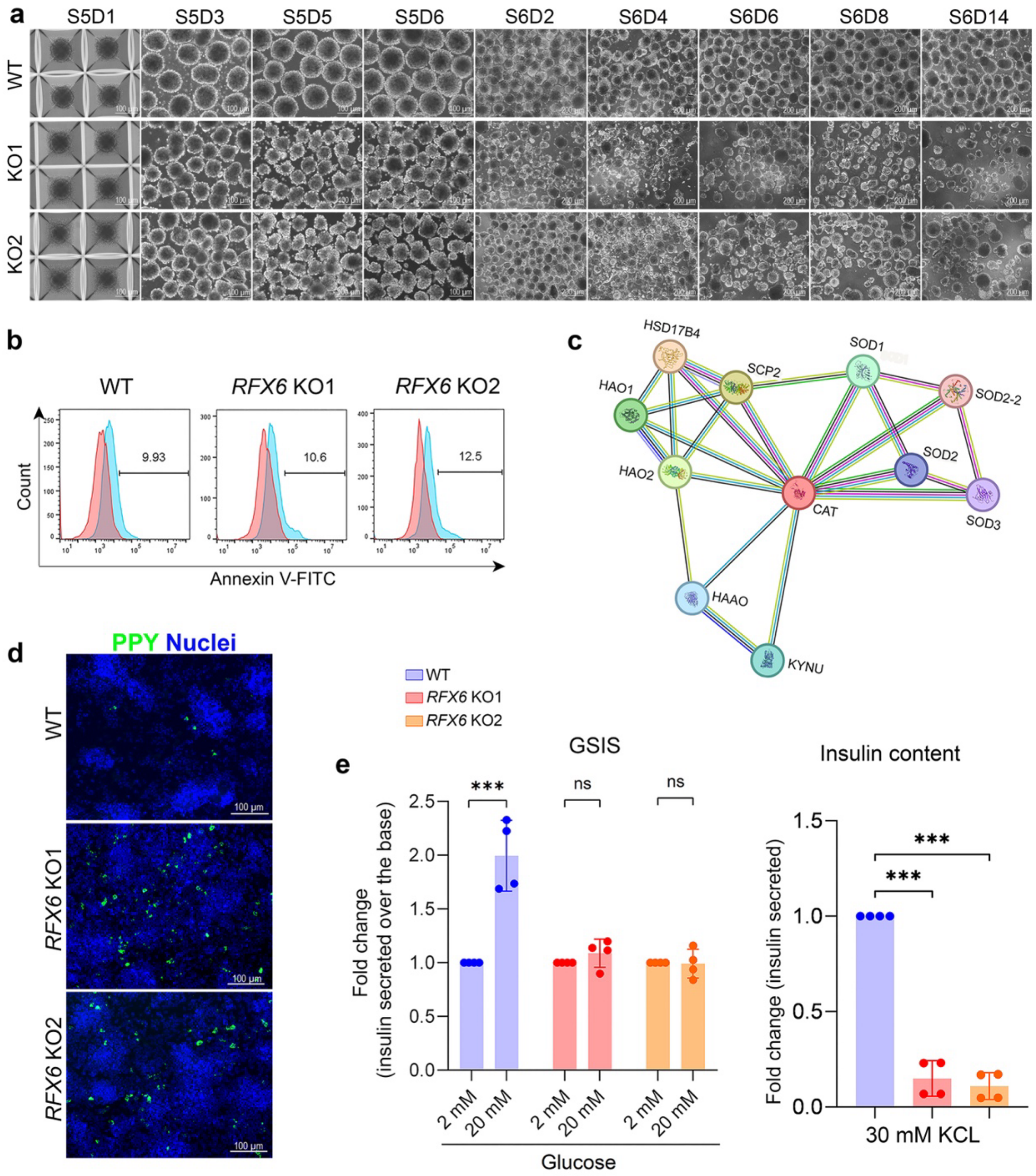

ESM Fig 6. Effect of RFX6 deficiency on pancreatic islet organoid development and functionality. (A) Morphological differences of pancreatic organoids generated from two RFX6 KO iPSCs compared to WT-iPSCs during EPs and islet stages. (B) Flow cytometry analysis and quantification of apoptosis (Annexin V+ cells) of RFX6 KO islets in comparison to WT-islets. (C) STRING network analysis showing the interaction of Catalase (CAT) protein with other proteins. (D) Immunofluorescence images showing increased PPY expression in RFX6 KO islets compared to WT-islets. (E) GSIS assay reflecting the lack of response to glucose challenges in RFX6 KO islets compared to WT-islets. Total insulin content stimulated by 30 mM KCL showed significant decrease in RFX6 KO islets compared to WT-islets. The data are presented as mean ±SD. ***$p < 0.001$. Scale bars = 100 µm, 200 µm.
